# Synthesis of Polymeric Carbon Nitrides in a Low-Cost Moka Furnace for Photocatalytic Hydrogen Generation with Visible Light

**DOI:** 10.1021/acs.jchemed.5c00114

**Published:** 2025-06-03

**Authors:** Malte Petersen, Jonathan Bauschulte, Savannah Talledo, Konrad Hotzel, Michael Wark, Kalina Peneva, Stefan Bernhard, Timm Wilke

**Affiliations:** 1 Institute of Chemistry, Chemistry Education, 11233 Carl von Ossietzky University Oldenburg, Carl-von-Ossietzky-Str. 9-11, 26129 Oldenburg, Germany; 2 Department of Chemistry, 6612 Carnegie Mellon University, 4400 Fifth Avenue, Pittsburgh, Pennsylvania 15213, United States; 3 Center for Energy and Environmental Chemistry Jena II (CEEC Jena II), 9378 Friedrich-Schiller-University Jena, Lessingstraße 12, 07743 Jena, Germany; 4 Institute of Organic Chemistry and Macromolecular Chemistry, Friedrich Schiller University Jena, Lessingstraße 8, 07743 Jena, Germany; 5 Institute of Chemistry, Chemical Technology 1, 11233 Carl von Ossietzky University Oldenburg, Carl-von-Ossietzky-Str. 9-11, 26129 Oldenburg, Germany; 6 Jena Center of Soft Matter (JCSM), Friedrich-Schiller University Jena, Philosophenweg 7, 07743 Jena, Germany; 7 Center for Energy and Environmental Chemistry Jena (CEEC Jena), Friedrich-Schiller University Jena, Philosophenweg 7a, 07743 Jena, Germany

**Keywords:** High School, Introductory Chemistry, First-Year Undergraduate, General, Laboratory Instruction, Hands-On Learning, Manipulatives, Nanotechnology, Photochemistry

## Abstract

Photochemical reactions are constantly present in our everyday lives and play an increasingly important role in our society. One relevant aspect for the future is the conversion of light energy into chemical energy to produce hydrogen. This process not only allows teachers to address the social dimension of hydrogen as an energy carrier but also enables relevant school topics such as photochemistry, catalysis, nanomaterials, energy transfer, and redox reactions to be taught. In the series of experiments presented, the photocatalyst polymeric carbon nitrides can be synthesized in a self-build €8 low-cost moka muffle furnace. These photocatalysts can produce detectable amounts of hydrogen within 15 min under UV light in a system with EDTA as an electron donor and platinum as a co-catalyst. Adding the photosensitizer Proflavin, the process is also possible with visible light. To enhance the experiments in school, a sensitive and inexpensive detection method was developed. A hydrogen-sensitive film can be produced and enables the detection of small amounts of hydrogen in a classroom.

## Graphical abstract

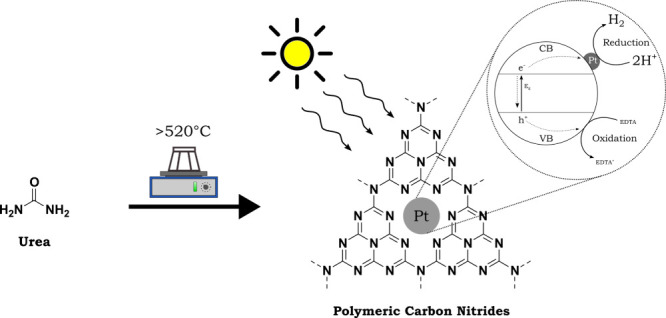

## Introduction

In many countries, alternatives to traditional fossil fuels are being explored as part of the global energy transition. Hydrogen is a promising candidate^1^ since it has the highest energy density among common fuels and can be efficiently converted into electricity using fuel cells. One way to produce hydrogen is through photocatalytic reactions, which have the advantage of using solar energy and do not require a large power grid, unlike electrolysis. Currently, most photocatalysts used in these processes are based on solid inorganic semiconductors of transition metal complexes. In science and science education alike, photocatalysts such as cadmium sulfide^2,3^ or zinc sulfide^4^ are often used. However, due to their toxic behavior and limited availability in recent years there has been a growing interest in organic materials as photocatalysts. In this context, Carbon Quantum Dots have often been discussed in chemistry education^5–7^ due to their properties as fluorescent markers. Nevertheless, their limitations in active sites for catalytic reactions make them unsuitable as photocatalysts. For this reason, Polymeric Carbon Nitrides (PCN), which provide more active sites for catalytic reactions due to their porous structure, have garnered increased interest to be promising candidates for this purpose.^8,9^

In this publication the authors have developed teaching materials and a series of experiments that aim to unlock the potential of PCN for hydrogen production in high school chemistry education (age 16 to 18). However, with minor adaptations, the material is also well-suited for use in undergraduate or graduate university courses. This paper presents (1) a low-cost muffle furnace that allows the synthesis of PCN as photocatalysts in an €8 moka pot. The obtained PCN can (2) produce hydrogen with UV light or even with visible light when a photosensitizer is added. Lastly, we present (3) a low-cost hydrogen-sensitive film which is based on the primary work of Talledo et al.^10^ to detect and track very small amounts of hydrogen in the liquid or gas phase. All experiments presented were previously examined and a detailed breakdown of these measurements, Notes for Instructors, and Student Handouts can be found in the section.

## Polymeric Carbon Nitrides Synthesis

A simple method to synthesize PCN is calcination. This method is typically carried out in a muffle furnace. However, these furnaces are often too expensive for schools, so we developed a low-cost alternative. In this bottom-up approach, the molecular precursor urea is heated in the moka-muffle furnace until the desired structure forms (Figure 1). When urea is heated to 520 °C, a reaction mechanism involving polycondensation and polyaddition reactions typically occurs.^11–13^ At temperatures above 240 °C, urea transforms into melamine, which further reacts at temperatures above 390 °C to form melem. This is followed by condensation into melon. At temperatures exceeding 520 °C, PCN are formed. In literature, this structure is mostly referred to as graphitic carbon nitride (g-C_3_N_4_).^14,15^ However, theoretical calculations suggest that this structure does not form completely, and a significant portion of melon remains in the structure.^13,16,17^ Therefore, the term polymeric carbon nitrides (PCN) is used instead of g-C_3_N_4_.

**Figure 1:**
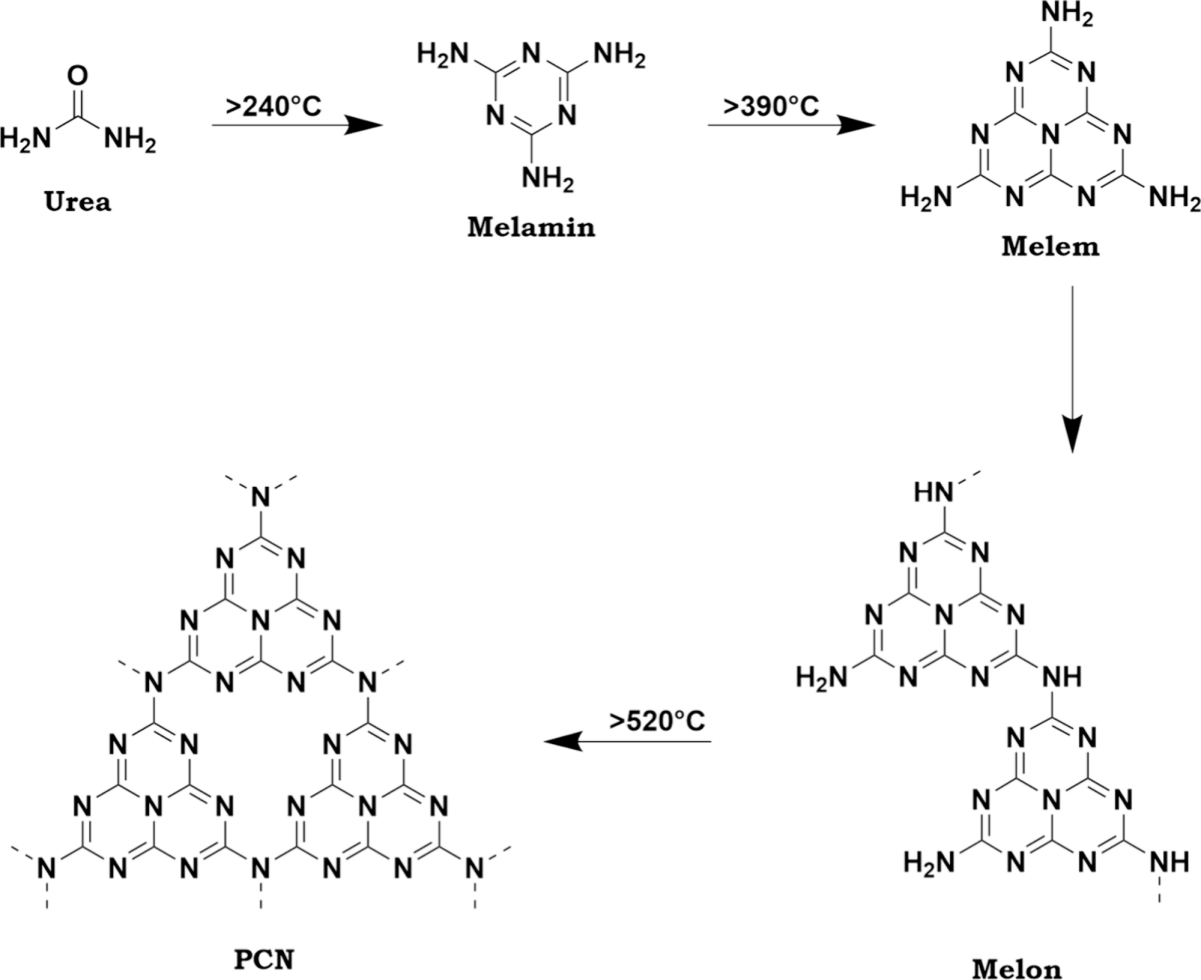
Schematic illustration of the formation process of PCN from urea.

### Experiment 1: PCN Synthesis

The PCN synthesis can be conducted with low-cost chemicals and equipment. Details on the construction and necessary equipment for the moka pot muffle furnace are described in the .

#### Equipment and Chemicals

Urea (CAS 57–13–6, C. Roth, €0.29/g), crucible with lid (25 mL), aluminum foil, hot plate, spatula, scale, homemade muffle furnace (), UV flashlight (λ = 395 nm).

#### Safety Note

The experiment should be conducted under a fume hood.

#### Procedure

Weigh out 4 g of urea into the crucible and place it, with the lid closed, in the muffle furnace which gets wrapped in aluminum foil (Figure 2). Set the hot plate to a temperature of 520 °C and heat the furnace for 150 min. Optionally, use a temperature probe to monitor the heating process externally, without opening the lid. Afterward, allow the setup to cool for 40 min, and store the product in a suitable container.

**Figure 2:**
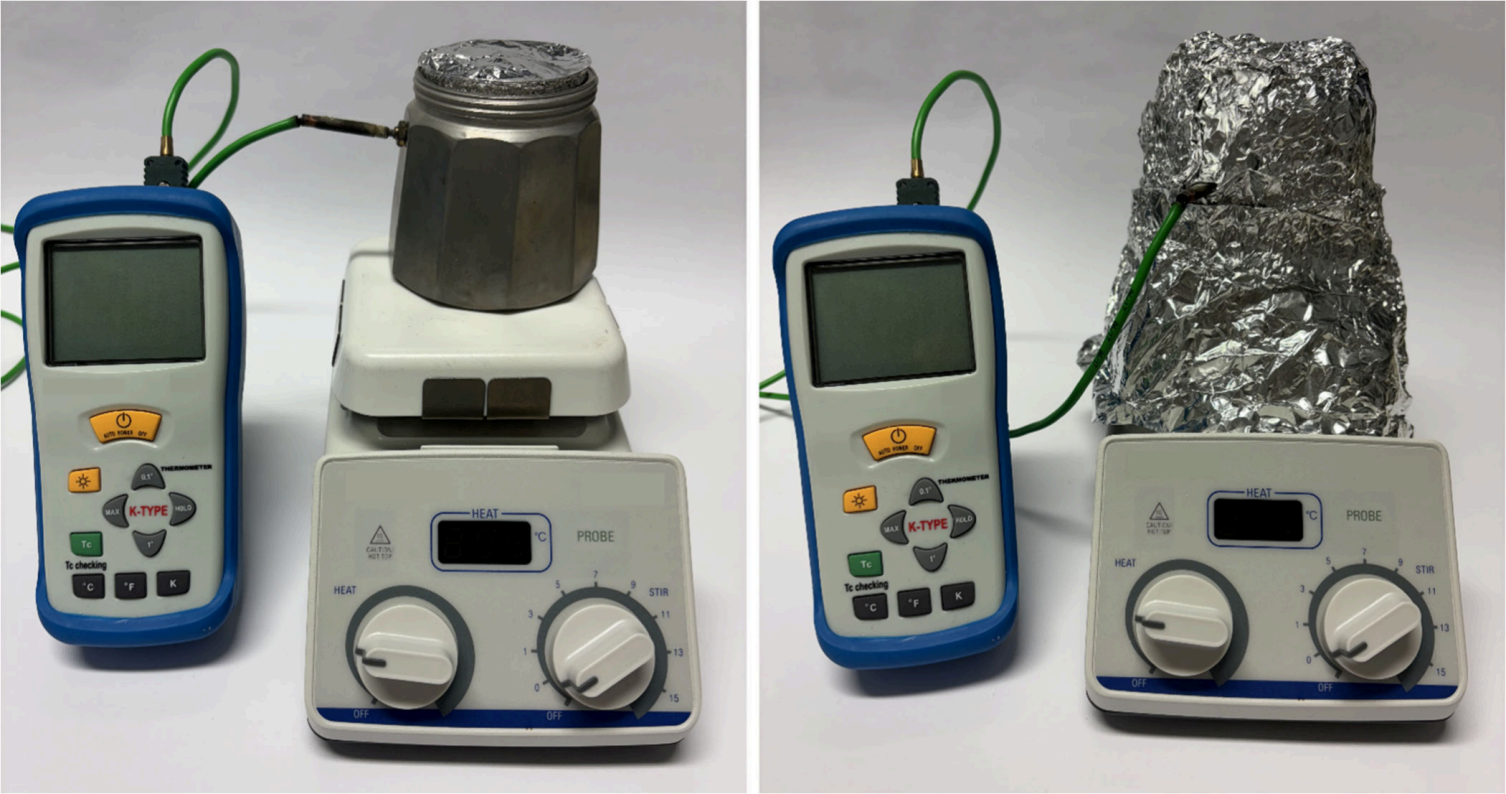
Assembled low-cost muffle furnace without (left) and with aluminum cladding (right).

#### Observation

During the heating process, smoke development and a pungent odor can be noticed. After cooling, a whitish-yellow solid is obtained, and the mass is reduced to around 260 mg. When the product is exposed to a UV flashlight, solid-state fluorescence can be observed (Figure 3).

**Figure 3:**
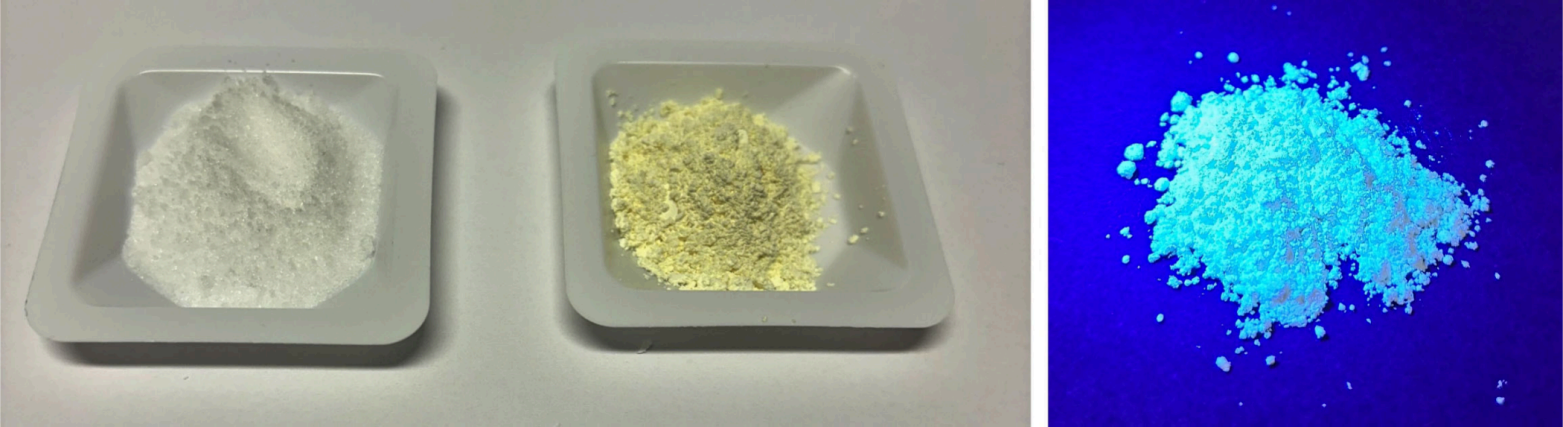
Comparison of the white reactant urea with the yellow product PCN under ambient light (left) and solid-state luminescence of the PCN under UV light (right).

#### Evaluation

The observed mass loss can be attributed to the side products carbon dioxide, water, and ammonia. The observation of fluorescence indicates the formation of PCN, which is due to the recombination of electrons from the conduction band to the valence band (Figure 4, step 5).^18^ The measurements for the PCN produced with the low-cost approach (see ) are consistent with values reported in the literature.^13,14^

**Figure 4:**
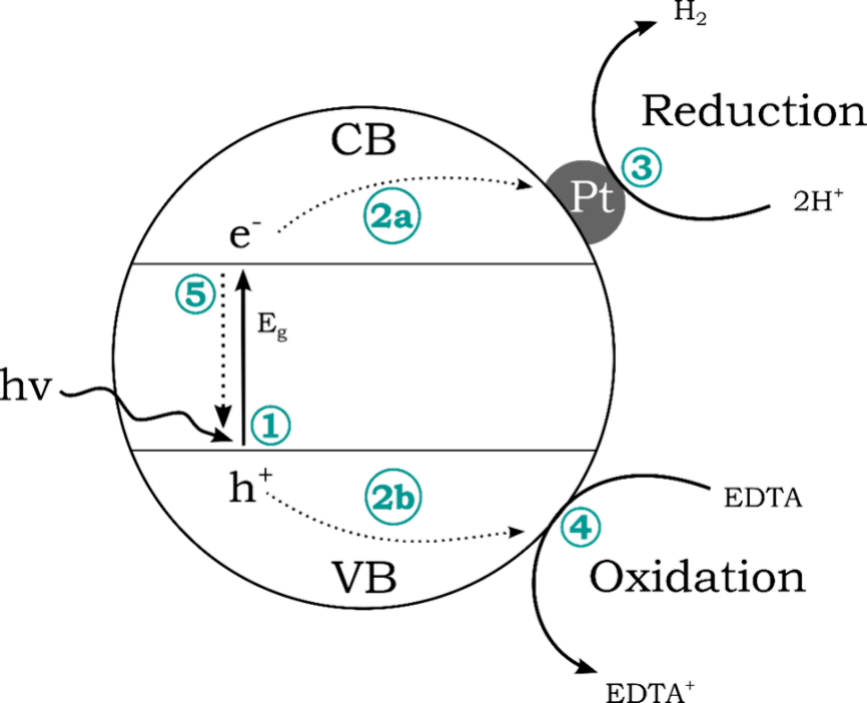
Schematic illustration of a photocatalytic process at a semiconductor.

#### Comment

The PCN can be stored for a long time. At the time of submission, samples produced six months earlier remained fully usable and showed no noticeable signs of degradation.

## Photocatalytic Generation of Hydrogen with PCN

In **heterogeneous** photocatalysis, incoming light with energy equal to or greater than the band gap (*hv*) is absorbed, exciting an electron from the valence band (VB) to the conduction band (CB) (Figure 4; (1), eq 1).^19^ This results in the formation of an electron–hole pair, where the hole remains in the valence band due to charge separation. The resulting charge carriers can then separate and migrate to the surface of the particle (step 2a/2b). At the surface, they can initiate a chemical reaction with an adsorbed species (steps 3 and 4). In this process, proton reduction to hydrogen (eq 2) and the oxidation of the electron donor ethylenediaminetetraacetic acid (EDTA) (eq 3) occur. The electron donor acts as a sacrificial reagent, enhancing the efficiency of the system. This is due to the lower oxidation potential of EDTA compared to the oxidation of water (eq 4). The surface characteristics of the photocatalyst are crucial for its function in chemical reactions. As PCN lack adequate active sites for hydrogen production,^20^ the cocatalyst platinum is employed to enable the reaction.^21^ During the process of photocatalysis, recombination of the excited electron and the hole can also occur (step 5), which is accompanied by a fluorescence phenomenon.^18^

$$\mathrm{PCN}\overset{hv}{\to }\mathrm{PCN}\left(e_{\mathrm{CB}}^{-}+h_{\mathrm{VB}}^{+}\right)$$ 1

$$2H^{+}+2e_{\mathrm{CB}}^{-}\to H_{2}↑$$ 2

$$\mathrm{EDTA}+h_{\mathrm{VB}}^{+}\to {\mathrm{EDTA}}^{+}$$ 3

$$2h_{\mathrm{VB}}^{+}+H_{2}O\to \frac{1}{2}O_{2}+2H^{+}$$ 4

In **photosensitized** photocatalysis, the electron for hydrogen production is not obtained directly from the valence band but through a preceding step. By using a photosensitizer (PS), it is possible to utilize light with a longer wavelength and thus lower the energy needed for photocatalytic hydrogen production. In general, photocatalytic hydrogen production is more efficient when driven by visible light rather than solely by UV light, as visible light makes up a larger portion of the solar spectrum, providing a more abundant and accessible energy source.

The process begins with the excitation of the photosensitizer by visible light (Figure 5, step 1, eq 5).^22^ In this step, an electron is promoted from the highest occupied orbital (HOMO) to the lowest unoccupied molecular orbital (LUMO). The excited electron can then migrate from the LUMO of the excited photosensitizer into the conduction band of the PCN (step 2, eq 6). After migrating to the platinum (step 3), a proton reduction to hydrogen can occur at the platinum surface, as in the previous photocatalysis process (step 4, eq 2). During the transfer of the electron to the PCN, the photosensitizer becomes oxidized (eq 6) and must be reduced again by the electron donor EDTA (eq 7) before a new cycle can start.

**Figure 5:**
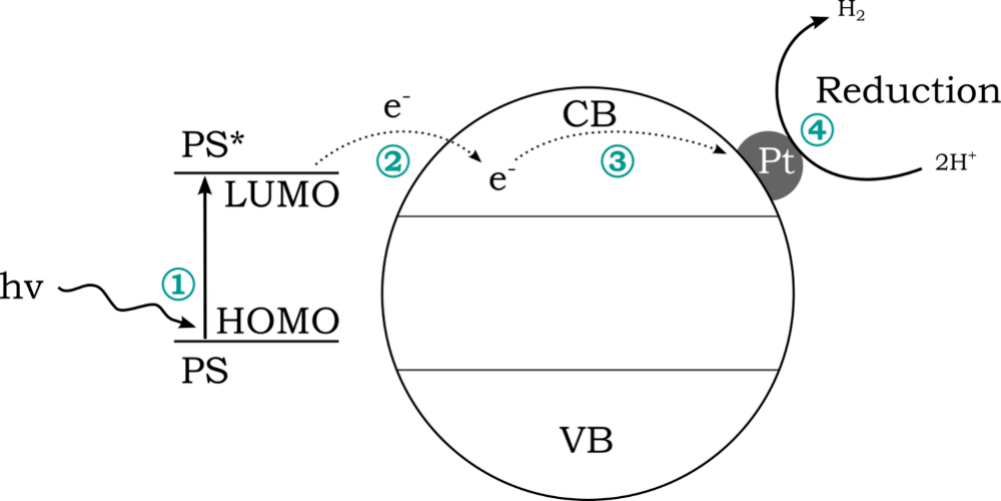
Schematic illustration of a photosensitized photocatalytic process at a semiconductor.

In these photocatalytic processes, electron transfer is only thermodynamically favorable if the LUMO of the excited species is energetically higher than the conduction band of the PCN.^22^ Various organic photosensitizers can be used in science to fulfill this condition, as these energy levels can easily be tailored by functionalization.^23,24^ In this system, the cheap and commercially available Proflavine is used, which was already used in different systems as a photosensitizer.^25–27^

$$\mathrm{PS}\overset{hv}{\to }{\mathrm{PS}}^{*}\left(e_{\mathrm{LUMO}}^{-}\right)$$ 5

$${\mathrm{PS}}^{*}\left(e_{\mathrm{LUMO}}^{-}\right)+\mathrm{PCN}\to {\mathrm{PS}}^{+}+\mathrm{PCN}\left(e_{\mathrm{CB}}^{-}\right)$$ 6

$${\mathrm{PS}}^{+}+\mathrm{EDTA}\to \mathrm{PS}+{\mathrm{EDTA}}^{+}$$ 7

### Experiment 2a: Photocatalytic Hydrogen Generation under UV Light

#### Equipment and Chemicals

UV flashlight (λ = 395 nm, Stier art.-no.: 95834653), hydrogen-sensitive film (diameter = 6 mm, thickness = 1 mm), glass vial with lid, pipettes, scale, spatula, stand, PCN (Exp. 1), platinum on alumina fiber (5% Pt, MBM, €15/g), EDTA solution (c = 4.9 mM, ROTH, CAS 6381–92–6, Merck, €0.81/g).

#### Procedure

4 mg of PCN, 3 mL of EDTA solution, and 5 mg of platinum-alumina are added to the glass vial (Sample #1). A small piece of hydrogen detection film is placed inside the jar, then sealed and gently shaken. Shake again if the film does not settle due to its similar density to water at the bottom. The UV flashlight is mounted vertically on a stand, and the sample is positioned above it for illumination. The exposure duration is 30 min.

#### Observation

After 15 min, the hydrogen-sensitive film shows a bluish color, progressing to complete darkening after 30 min (Figure 6). The darkening of the film indicates the generation of hydrogen (Exp. 3).

**Figure 6:**
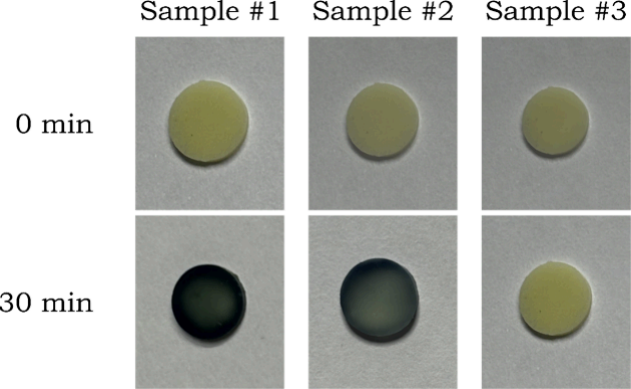
Coloration of the hydrogen detection films after 30 min of irradiation. Sample #1: PCN, EDTA, platinum under UV light. Sample #2: PCN, EDTA, proflavine, platinum under blue light. Sample #3: PCN, EDTA, platinum under blue light.

#### Evaluation

By illuminating the PCN, photochemical hydrogen production occurs under UV light with the aid of a cocatalyst, as confirmed by gas chromatography ().

#### Purification

The platinum-alumina fibers can be removed from the solutions using tweezers and reused after annealing with a gas burner. The PCN can be washed with water and reused for another photochemical process after drying.

### Experiment 2b: Photocatalytic Hydrogen Generation under Visible Light

#### Equipment and Chemicals

Flashlights with blue light (Dask Fire HQM-01) or regular flashlight with blue filter, hydrogen-sensitive film (diameter = 6 mm, thickness = 1 mm), glass vials with lids, pipettes, scale, spatula, stand, PCN (Exp. 1), platinum on alumina fiber (5% Pt, MBM, €15/g), EDTA solution (c = 4.9 mM, (ROTH, CAS 6381-92-6, Merck, €0.81/g)), stock solution (EDTA 4.9 mM, proflavine 0.05 mM (CAS 1811-28-5; Merck, €5.92/g)).

#### Procedure

In a glass vial, 3 mL of the stock solution, 4 mg of PCN, and 5 mg of platinum-alumina are added (Sample #2). For Sample #3, the EDTA solution is used instead of the stock solution. A small piece of hydrogen detection film is placed inside each jar, sealed, and shaken gently. If the film does not settle at the bottom, shake again. The two flashlights are mounted vertically in a stand, and the samples are positioned above them. The illumination time with blue light is 30 min.

#### Observation

After 30 min, the film in Sample #3 shows no coloration. However, in Sample #2, the film turns blue (Figure 6).

#### Evaluation

By using a photosensitizer, a broader spectrum of light can be utilized, enabling hydrogen production under blue light (Sample #2). Control experiments carried out in the absence of the photosensitizer showed that no hydrogen was generated (Sample #3). All results were verified by gas chromatography (). In a similar experiment reported by Kremer and Tausch (2021), several of the same chemicals were used (EDTA, proflavine, and 5% Pt@alumina fibers).^28^ While proflavine served as a photocatalyst in their study, we employ it as a photosensitizer.

#### Purification

See experiment 2a

## Hydrogen Sensing Film

While the above-described reaction will produce enough hydrogen for a successful squeaky pop test (oxyhydrogen test) at a given point in time, it is convenient to have an easy method ready at hand that detects even small amounts of hydrogen produced in (short) school lesson settings. The hydrogen-sensitive film presented in the following is based on preliminary work from Talledo et al.^10^ The aim is to complement existing detection methods, such as the mentioned (qualitative) pop test, the Heterogeneous Catalyzed Hydrogen Test (HeCHT)^29^ or low-cost hydrogen sensors.^4,30^

The fabricated films are composed of polydimethylsiloxane (PDMS) and incorporate 0.05% (ω/ω) platinum-loaded tungsten­(VI) oxide as the active material for hydrogen sensing. Detection relies on the hydrogen spillover mechanism, a phenomenon previously explored by Adams and Chen.^31^ In this process, hydrogen molecules dissociate on the platinum catalyst surface, which then migrate into the metal oxide structure. The presence of hydrogen atoms within the oxide leads to the formation of deep blue tungsten bronze.^32^ This visible color shift is linked to the generation of W^5+^ ions, resulting in a mixture of +V and +VI oxidation states and enabling intervalence charge transfer (IVCT) transitions in the visible light range.^33,34^

If the darkly colored film is left in the air for a day, the favored reverse reaction occurs, and the film discolors again due to the atmospheric oxygen (Figure 7). The film can then be used again to detect hydrogen.

**Figure 7:**
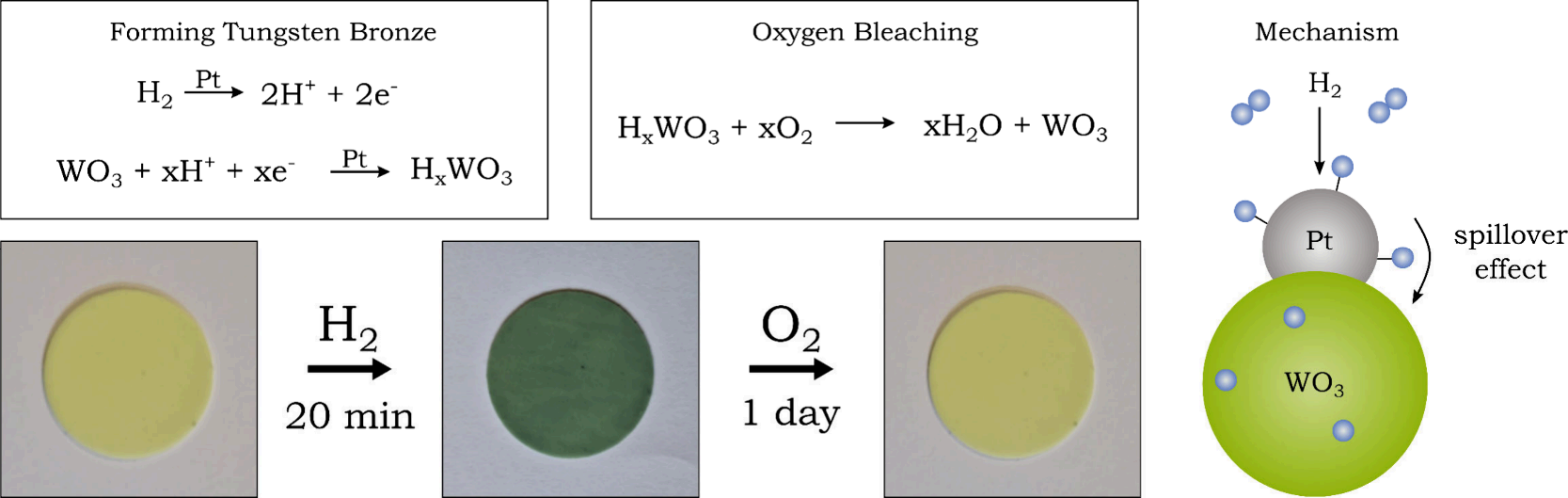
Left: Reaction equation for coloration and decolorization of the detection film. Right: Spillover mechanism for the formation of deep blue tungsten bronze.

### Experiment 3: Testing the Hydrogen Sensitive Film

The procedure to synthesize the hydrogen sensitive film is described in detail in the .

#### Chemicals and Materials

Hydrogen-sensitive film (diameter = 15 mm, thickness = 1 mm), Erlenmeyer flasks with stoppers, gas cylinders with oxygen, carbon dioxide, nitrogen, hydrogen.

#### Procedure

A piece of hydrogen-sensitive film is placed in each Erlenmeyer flask. Each flask is flushed with either oxygen, carbon dioxide, nitrogen, or hydrogen, and immediately sealed.

#### Observation

After 3 min, a dark coloration of the film is observed in the hydrogen atmosphere. This coloration intensifies over time. The other pieces of the detection film do not color in the different atmospheres, even after an extended period.

#### Evaluation

Upon contact with hydrogen, the blue tungsten bronze forms, as shown in Figure 7, and the film becomes colored. When the colored film is exposed to ambient air, it gradually fades back to yellow within one to two days, depending on the intensity of the coloration, and can be reused.

## Safety Information

A lab coat and protective goggles should be always worn during the experiments. In experiment 1 the synthesis must be carried out under a fume hood due to the toxic intermediate product melamine (harmful to health) and the formation of low amounts of ammonia (corrosive, toxic, environmental hazard) during the heating process. In experiment 2a/b, EDTA (serious health hazard) is used as an electron donor in a low concentration which is safe to use at school. Experiment 3 involves working with different gas cylinders and should be carried out by a trained person.

## Educational Setting and Implementation

Following the development of the presented experiments, we arranged an out-of-school laboratory (OSL) course with accompanying teaching materials (two theory stations T1, T2 and three experimental stations E1, E2, E3) that align with current scientific research developments and provide an opportunity to explore the potential of PCN as nanomaterials and their reactions with light through simple experiments. The topics addressed in these experiments and the accompanying materials align with the core chemistry concepts taught in higher education within the German educational system. Examples include the synthesis of nanomaterials (structure–property concept, theory station T1), fundamental photochemical processes (energy concept, theory station T2), and redox reactions (donor–acceptor concept, experiments E1–E3). All stations with a more detailed description of the materials and learning goals can be found in the . If any reader is interested in a realization, samples of the detection film can be requested from the corresponding authors.

The aim of the two theory stations () is to enable students to evaluate the experiments. Therefore, we introduce the basics of synthesizing nanomaterials and the concept of photocatalysts. The learning goal of the first station is for the students to be able to explain bottom-up and top-down syntheses for organic nanomaterials and apply them to the synthesis of PCN (corresponding to Exp. 1 in this paper). After the subsequent second theory station, students should be able to describe the relevant steps of a heterogeneous photocatalytic reaction with the aid of an illustration. In an experiment (corresponding to Exp. 3), the students learn that the coloration of the film is due to the presence of hydrogen. This is necessary to confirm the formation of hydrogen in the following experiments (corresponding to Exp. 2a, 2b). Here, students can apply their previous knowledge of the theory stations, explain the specific process for the photocatalytic production of hydrogen, and learn how a photosensitizer works. Based on the findings, a discussion about its current and future significance for society appears appropriate. An in-detail description of the underlying (photochemical) processes that take place is not to be expected in school but can be added for high-achieving students or seminar projects.

The presented materials and experimental procedures are also suitable for implementation in diverse educational contexts. By employing inexpensive reagents and readily available materials to construct a low-cost muffle furnace, we aimed to ensure broad accessibility and adaptability across different countries and institutional settings. The core competencies, particularly in the fields of nanotechnology, redox chemistry, and photocatalysis, are relevant to school but also university-level curricula in many countries. Furthermore, the materials can be tailored to varying educational levels by modulating the complexity of the evaluation methods or the analytical depth, such as the optional integration of gas chromatography for hydrogen detection.

### Ethical Statement

The collection and use of data comply with the European General Data Protection Regulation as well as all relevant national and university ethical guidelines. Participation in the study is entirely voluntary. Guardians of underage students were informed in advance, and written consent was obtained – either from the guardians or, in the case of students over 18, from the students themselves. All data is collected and stored anonymously. To ensure this, each participant generates a personal code at the beginning of the study. After completion, the data cannot be traced back to individuals unless participants choose to exercise their data protection rights and voluntarily disclose their code.

### Pilot Study and Evaluation

According to research framework proposed in the model of educational transfer research,^35^ the conceptualized experiments and materials were piloted, evaluated, and optimized before dissemination into school practice. The experiments were conducted in an OSL where school students (*N* = 25) from the 11th grade of a German high school (aged 16–17) visited the university for the course. For the evaluation of the feasibility of the experiments, we focused on the core experiments during the pilot study, evaluating hydrogen generation under UV light and the use of the detection film with a questionnaire containing three quantitative items on a five-point Likert scale (Figure 8).

**Figure 8:**
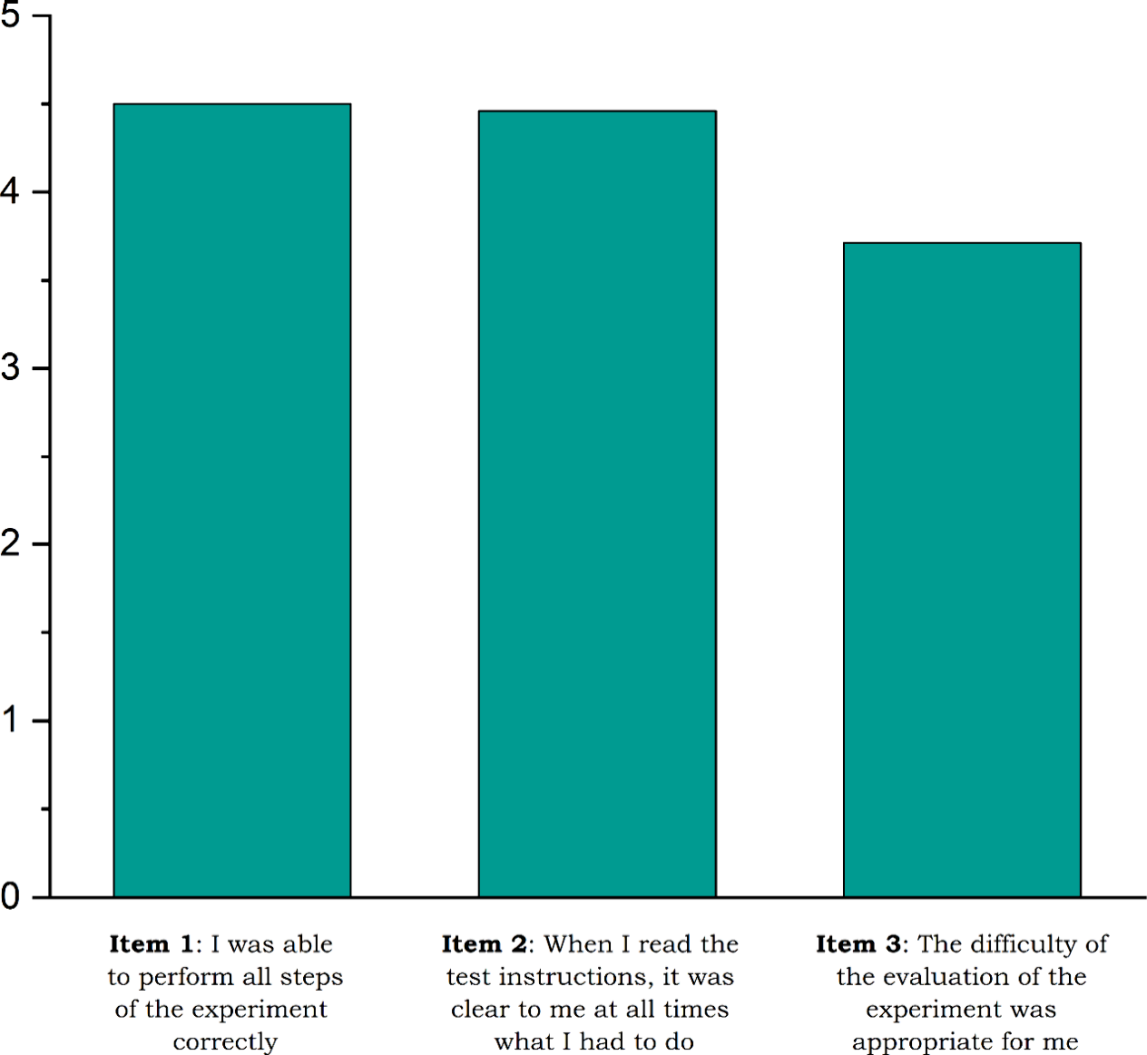
Mean values of items 1–3 in the survey (*N* = 24) regarding the manageability of the experiment.

The results show that the students managed the experiment well (item 1, M = 4.50) and found the instructions clear (item 2, M = 4.46). The related evaluation tasks were also handled effectively, according to the students’ feedback (item 3, M = 3.71).

In an additional qualitative item (4), the students were asked to describe what they had learned from this experimental unit. The responses indicated that they were particularly intrigued by the new method of hydrogen detection (11 mentions), with the aspects of photocatalytic hydrogen production being mentioned afterward (10 mentions).

To dive deeper into the qualitative feedback, we conducted an interview with four different students. The aim of this was to get directly an impression about the feasibility of the experiment () and what they felt was the most important topic they had learned (). All participants reported that they had no trouble with the experiment or the evaluation tasks (with the support provided). They understood the connection between the blue coloration and the presence of hydrogen, which helped them in analyzing the photocatalysis process.

The study demonstrates that students are capable of using a simple photocatalytic system for hydrogen production. The detection film enabled the experiment to be conducted without expensive or complex analytics. The PCN produced in the low-cost muffle furnace proved to be highly functional in this trial.

## Summary and Outlook

This series of experiments allows teachers to utilize the potential of photochemical processes in the classroom. By using an €8 moka muffle furnace, there is a low-cost and noncomplex way to synthesize a potent photocatalyst. By developing the hydrogen detection film, we enhance the possibility of tracking photochemical hydrogen production without complex analysis. Overall, a series of experiments was presented that addresses a subject area that will become increasingly important in the future. By linking the generation of photocatalytic hydrogen with the topic of nanomaterials, a promising learning subject for school education has been opened. The initial feedback from students shows a positive attitude toward the topic, which needs to be analyzed and addressed in more detail in further work. Future research will furthermore expand the possibilities of photocatalytic processes with PCN. Additionally, the use of hydrogen detection film will be explored in other classic school experiments. Based on this, the materials will be further optimized for integration into school and university education.

## Supplementary Material











## Notes

Notes for Instructors for the Student Materials, Construction of the Moka Muffle Furnace, Synthesis of the Detection Film, Measurements and Characterization, and interviews (PDF)
Student Handouts (PDF, DOCX)
Student Handouts Solutions (PDF, DOCX)

## References

[1] IEA . The Future of Hydrogen:Seizing today’s opportunities. https://www.iea.org/reports/the-future-of-hydrogen#overview (accessed 2024–11–20).

[2] Sahin M., Koca A. (2003). Photocatalytic Hydrogen Production by Direct Sunlight: A Laboratory
Experiment. J. Chem. Educ..

[3] Li X., Deng Y., Jiang Z., Shen R., Xie J., Liu W., Chen X. (2019). Photocatalytic
Hydrogen Production over CdS Nanomaterials:
An Interdisciplinary Experiment for Introducing Undergraduate Students
to Photocatalysis and Analytical Chemistry. J. Chem. Educ..

[4] Maaß M. C., Tasch A., Jooss C., Waitz T. (2022). Photocatalytic Hydrogen
Evolution Using ZnS Particles and LEDs. J. Chem.
Educ..

[5] Schneider E. M., Bärtsch A., Stark W. J., Grass R. N. (2019). Safe One-Pot Synthesis
of Fluorescent Carbon Quantum Dots from Lemon Juice for a Hands-On
Experience of Nanotechnology. J. Chem. Educ..

[6] Pham S. N., Kuether J. E., Gallagher M. J., Hernandez R. T., Williams D. N., Zhi B., Mensch A. C., Hamers R. J., Rosenzweig Z., Fairbrother H., Krause M. O., Feng Z. V., Haynes C. L. (2017). Carbon
Dots: A Modular Activity To Teach Fluorescence
and Nanotechnology at Multiple Levels. J. Chem.
Educ..

[7] Wu F., Zhang R., Zhou J. (2024). Shrimp-Shell-Derived Carbon Dots
for Quantitative Detection by Fluorometry and Colorimetry: A New Analytic
Chemistry Experiment for University Education. J. Chem. Educ..

[8] Alaghmandfard A., Ghandi K. (2022). A Comprehensive Review
of Graphitic Carbon Nitride
(g-C3N4)-Metal Oxide-Based Nanocomposites: Potential for Photocatalysis
and Sensing. Nanomaterials (Basel, Switzerland).

[9] Iqbal O., Ali H., Li N., Al-Sulami A. I., F Alshammari K., Abd-Rabboh H. S., Al-Hadeethi Y., Din I. U., Alharthi A. I., Altamimi R., Zada A., Wang Z., Hayat A., Zahid Ansari M. (2023). A review on
the synthesis, properties, and characterizations
of graphitic carbon nitride (g-C3N4) for energy conversion and storage
applications. Materials Today Physics.

[10] Talledo S., Kubaney A., Baumer M. A., Pietrak K., Bernhard S. (2024). High throughput
methodology for investigating green hydrogen generating processes
using colorimetric detection films and machine vision. Digital Discovery.

[11] Zheng Y., Liu J., Liang J., Jaroniec M., Qiao S. Z. (2012). Graphitic carbon
nitride materials: controllable synthesis and applications in fuel
cells and photocatalysis. Energy Environ. Sci..

[12] Thomas A., Fischer A., Goettmann F., Antonietti M., Müller J.-O., Schlögl R., Carlsson J. M. (2008). Graphitic carbon
nitride materials: variation of structure and morphology and their
use as metal-free catalysts. J. Mater. Chem..

[13] Weers M., von Seggern A. R., Vocke H., Taffa D. H., Wark M. (2024). Two Ways to
more NH 2 -Groups: Formation of Polymeric Carbon Nitride via Melem
Tetramer Nano Sheets or Supramolecular Assembly of Melamine and Cyanuric
Acid for Applications as Photocatalyst. ACS
Appl. Nano Mater..

[14] Wen J., Xie J., Chen X., Li X. (2017). A review on g-C 3 N 4 -based photocatalysts. Appl. Surf. Sci..

[15] Molaei M. J. (2023). Graphitic
carbon nitride (g-C3N4) synthesis and heterostructures, principles,
mechanisms, and recent advances: A critical review. Int. J. Hydrogen Energy.

[16] Tyborski T., Merschjann C., Orthmann S., Yang F., Lux-Steiner M.-C., Schedel-Niedrig T. (2013). Crystal structure of polymeric carbon nitride and the
determination of its process-temperature-induced modifications. J. Phys.: Condens. Matter.

[17] Im C., Kirchhoff B., Krivtsov I., Mitoraj D., Beranek R., Jacob T. (2023). Structure
and Optical Properties of Polymeric Carbon Nitrides from
Atomistic Simulations. Chem. Mater..

[18] Qu B., Sun J., Li P., Jing L. (2022). Current advances on g-C3N4-based
fluorescence detection for environmental contaminants. Journal of hazardous materials.

[19] Li J., Wu N. (2015). Semiconductor-based
photocatalysts and photoelectrochemical cells
for solar fuel generation: a review. Catal.
Sci. Technol..

[20] Pan Z., Zheng Y., Guo F., Niu P., Wang X. (2017). Decorating
CoP and Pt Nanoparticles on Graphitic Carbon Nitride Nanosheets to
Promote Overall Water Splitting by Conjugated Polymers. ChemSusChem.

[21] Wang X., Maeda K., Thomas A., Takanabe K., Xin G., Carlsson J. M., Domen K., Antonietti M. (2009). A metal-free
polymeric photocatalyst for hydrogen production from water under visible
light. Nature materials.

[22] Gonuguntla S., Kamesh R., Pal U., Chatterjee D. (2023). Dye sensitization
of TiO2 relevant to photocatalytic hydrogen generation: Current research
trends and prospects. Journal of Photochemistry
and Photobiology C: Photochemistry Reviews.

[23] Knorr G., Hotzel K., Chettri A., Skabeev A., Wächtler M., Dietzek-Ivanšić B., Peneva K. (2023). Unlocking the potential
of ketocoumarins: efficient photosensitizers for sustainable light
driven hydrogen evolution. J. Mater. Chem. A.

[24] Costabel D., Nabiyan A., Chettri A., Jacobi F., Heiland M., Guthmuller J., Kupfer S., Wächtler M., Dietzek-Ivanšić B., Streb C., Schacher F. H., Peneva K. (2023). Diiodo-BODIPY Sensitizing
of the Mo3S132- Cluster for
Noble-Metal-Free Visible-Light-Driven Hydrogen Evolution within a
Polyampholytic Matrix. ACS Appl. Mater. Interfaces.

[25] Ghosh T., Slanina T., König B. (2015). Visible light photocatalytic reduction
of aldehydes by Rh­(iii)-H: a detailed mechanistic study. Chemical science.

[26] Pileni M. P., Graetzel M. (1980). Light-induced redox
reactions of proflavine in aqueous
and micellar solution. J. Phys. Chem..

[27] Kalyanasundaram K., Dung D. (1980). Role of proflavin as a photosensitizer for the light-induced hydrogen
evolution from water. J. Phys. Chem..

[28] Kremer R., W. Tausch M. (2021). Hydrogen Goes Green - Model Experiments for Artificial
Photosynthesis. WJCE.

[29] Reinmold M., Lühken A. (2023). Rethinking
the Squeaky Pop Test–A Novel Hydrogen
Test for Chemistry Classes. J. Chem. Educ..

[30] Petersen M., Worliczek P., B. Max J., Nabiyan A., Wejner M., Eichhorn J., Streb C., H. Schacher F., Wilke T. (2021). Hydrogen Evolution Reaction with Sunlight for School Chemistry Education. WJCE.

[31] Adams B. D., Chen A. (2011). The role of palladium in a hydrogen economy. Mater. Today.

[32] Cui Y., Liang F., Ji C., Xu S., Wang H., Lin Z., Liu J. (2019). Discoloration Effect and One-Step Synthesis of Hydrogen
Tungsten and Molybdenum Bronze (H x MO3) using Liquid Metal at Room
Temperature. ACS omega.

[33] Chen L., Cooper A. C., Pez G. P., Cheng H. (2007). Mechanistic Study on
Hydrogen Spillover onto Graphitic Carbon Materials. J. Phys. Chem. C.

[34] Bérubé V., Radtke G., Dresselhaus M., Chen G. (2007). Size effects on the
hydrogen storage properties of nanostructured metal hydrides: A review. Int. J. Energy Res..

[35] Fruntke A., Behnke M., Stafast L. M., Träder T., Dietel E., Vollrath A., Weber C., Schubert U. S., Wilke T. (2023). Targeted Drug Delivery: Synthesis
of Smart Nanocarriers for School
Chemistry Education. J. Chem. Educ..

