# Supplementary material for: Synthesis of Polymeric Carbon Nitrides in a Low-Cost Moka Furnace for Photocatalytic Hydrogen Generation with Visible Light
Source: J Chem Educ. 2025 Jun 3;102(7):2912–9. doi: 10.1021/acs.jchemed.5c00114 (PMC12243077; doi:10.1021/acs.jchemed.5c00114)
Supplement: Supplementary file 1 [file ed5c00114_si_001.pdf]

Supporting Information

Synthesis of Polymeric Carbon Nitrides in a low-cost Moka Furnace for Photocatalytic Hydrogen Generation with Visible Light

Malte Petersen[1,*], Jonathan Bauschulte[1], Savannah Talledo[2], Konrad Hotzel[3,4], Michael Wark[5], Kalina Peneva[4,6,7], Stefan Bernhard[2], Timm Wilke[1,6,*]

[1] Institute of Chemistry, Chemistry Education, Carl von Ossietzky University Oldenburg, Carl-von-Ossietzky-Str. 9-11, 26129 Oldenburg, Germany

[2] Department of Chemistry, Carnegie Mellon University, 4400 Fifth Avenue, Pittsburgh, Pennsylvania 15213, USA

[3] Center for Energy and Environmental Chemistry Jena II (CEEC Jena II), Friedrich-Schiller-University Jena, Lessingstraße 12, 07743 Jena, Germany

[4] Institute of Organic Chemistry and Macromolecular Chemistry, Friedrich Schiller University Jena, Lessingstraße 8, 07743 Jena, Germany

[5] Institute of Chemistry, Chemical Technology 1, Carl von Ossietzky University Oldenburg, Carl-von-Ossietzky-Str. 9-11, 26129 Oldenburg, Germany

[6] Jena Center of Soft Matter, Friedrich-Schiller University Jena, Philosophenweg 7, 07743 Jena, Germany

[7] Center for Energy and Environmental Chemistry Jena (CEEC Jena), Friedrich-Schiller University Jena, Philosophenweg 7a, 07743 Jena, Germany

TABLE OF CONTENTS

Page	Title
2	NOTES FOR INSTRUCTORS FOR THE STUDENT MATERIALS
3	CONSTRUCTION OF THE MOKA MUFFLE FURNACE
5	SYNTHESIS OF THE DETECTION FILM
5	SAFETY INFORMATION
6	MEASUREMENTS AND CHARACTERIZATION
9	INTERVIEWS
10	AUTHOR INFORMATION
11	ACKNOWLEDGMENTS
11	REFERENCES

NOTES FOR INSTRUCTORS FOR THE STUDENT MATERIALS

The materials in the associated documents were tested in a three-hour student laboratory setting. The students (aged 16-18) came from a German gymnasium. Before the two theory stations were carried out, a general introduction was given to activate the necessary scientific basics. The problem of a society based on fossil fuels was also addressed. The long-term finite resources and climate change promoted by burning these energy sources allow a transition to the sun as a future alternative energy source. With this goal in mind, the pupils start the school laboratory. After each station, their results were collected in plenary and compared with each other. Furthermore, there was lively discussion with the students during the student lab and the groups were supported in completing the tasks. All students worked in groups of two to three.

Specific Educational Goals for each station:

T1: The students can explain the bottom-up and top-down principles for synthesizing organic nanomaterials and assign exemplary processes to these categories.

T2: Students can describe the relevant steps of a photocatalytic process with the aid of an information text and a diagram.

E1: The students can explain the coloration of the detection film to a reaction with hydrogen and attribute this to the formation of tungsten bronze.

E2: The students can set up the reaction equations that take place during photocatalytic hydrogen production and explain the process with the aid of a diagram.

E3 (advanced): With the help of a diagram, the students can explain the photosensitized hydrogen generation and discuss its significance for a future society.

Prior knowledge of the students:

The students had already been introduced to the subject area of photoluminescence by the teacher before the student laboratory. They were able to explain fluorescence and phosphorescence with the aid of a simplified Jablonski diagram. They were also familiar with the topics of redox reactions and how a classic catalyst works from their regular lessons.

Preparation before the student laboratory:

Before the student laboratory, the polymeric carbon nitrides were synthesized in the low-cost muffle furnace (Manuscript, experiment 1), as this cannot be easily realized in the actual time period of a student laboratory of three hours. Furthermore, the detection film was synthesized (SI, Synthesis of the Detection Film) and punched into small pieces. Should an interested reader wish to implement this, they can contact the corresponding authors for a supply of the detection film.

CONSTRUCTION OF THE MOKA MUFFLE FURNACE

The lower part of the espresso maker (Fig. S1, A), consisting of the base and the sieve insert (B, C), is used as a reaction vessel. For this the extended tube of the sieve should be shortened with a saw. The upper part is not needed further. To use the muffle furnace, the reaction vessel is insulated with aluminum. To do this, the sieve insert is first filled with aluminum foil and then fully wrapped with a thin layer (D, E). In the next step, a piece of aluminum foil is folded several times and shaped into a circle so that it fits into the base of the espresso maker without protruding (G). The aluminum foil around the valve is pressed in slightly to leave the valve exposed. Now, a crucible with the desired reactant can be placed inside (G), and the reaction vessel can be sealed with the insulated sieve insert. In the next step, the base is placed on a hot plate (H), and the optional temperature sensor is inserted through the valve from the outside to the inside (I). Afterwards, the surface of the hot plate, along with the reaction vessel, is wrapped with aluminum foil (J). It is essential to ensure that the base of the temperature sensor is well insulated. To use the muffle furnace, the hot plate is turned on and the temperature is monitored with the measuring device.

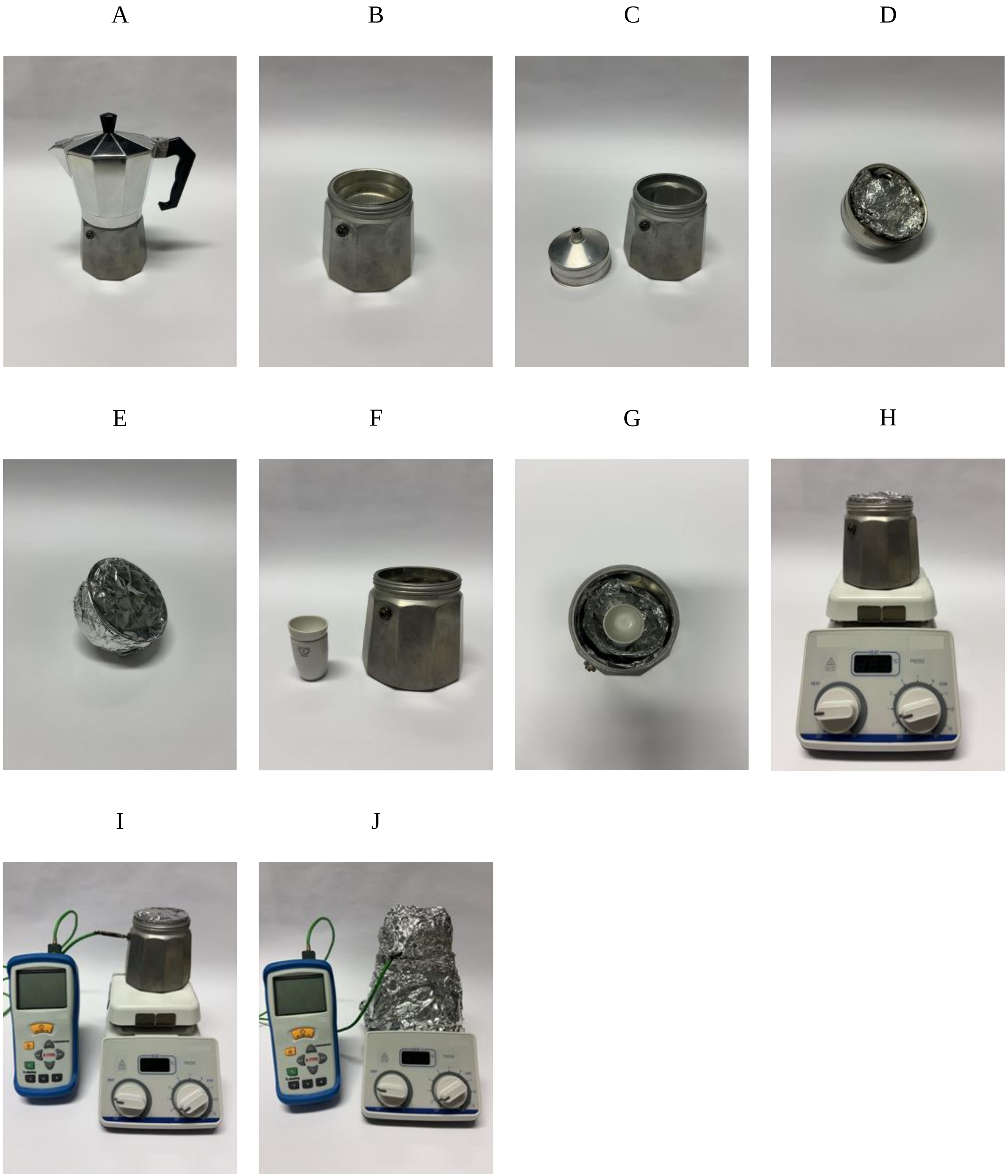

Figure S1. Visualized process of building the Moka Muffle oven.

SYNTHESIS OF THE DETECTION FILM

Sensing Material Synthesis

Materials: potassium tetrachloroplatinate (CAS 10025-99-7, fisher scientific, €165/g), tungsten(VI) oxide (CAS 1314-35-8, fisher scientific, €7,72/g), sodium hydroxide (CAS 1310-73-2, ROTH, €35/kg), sodium borohydride (CAS 16940-66-2, fisher scientific, €0,7/g), dest. water, beaker, magnetic stirrer, stirring rod, spatula, balance, desiccator, filter paper, büchner funnel.

Procedure:

The synthetic procedure was adapted from previous publications.[1,2] 10 mg of potassium tetrachloroplatinate was added to a 200 mL aqueous suspension containing 20 g of tungsten(VI)oxide. This mixture was vigorously stirred at room temperature for two hours. Then, 3 g of sodium hydroxide and 1 g of sodium borohydride were added, and the mixture was stirred for an additional two hours at room temperature. The resulting powder was filtered, washed with water (2 × 10 mL), and dried under vacuum overnight.

Experiment – Integration into PDMS Film

Materials: n-Octyltriethoxysilane (97%, CAS 2943-75-1, fisher scientific, €1,04/mL), methanol (HPLC Gradient Grade, CAS 67-56-1, ROTH, €20/L), Sylcap™ 284-F (MicroLubrol, $189), sensing material, borosilicate glass, stainless-steel strips, oven, scale, spatula, plastic beaker.

Procedure: 360 mg of hydrogen-sensitive powder, 110 µL of OTS (n-octyltriethoxysilane), and 3 mL of methanol are added to a plastic beaker and stirred vigorously for ten minutes or until only a minimal amount of methanol remained and no large solid particles were left. A commercially available PDMS kit, Sylcap™ 284-F, is used to suspend the hydrogen-sensing material. 1 g of curing agent and 9 g of elastomer base are added to the beaker and stirred until the powder was evenly distributed and the color uniform. The mixture was then poured evenly onto a borosilicate glass surface bordered by 1 mm thick stainless-steel strips. The films were cured at 80°C for 1 hour in an oven.

SAFETY INFORMATION

The synthesis of the sensing film is a teacher experiment and must not be carried out by students. The chemicals potassium tetrachloroplatinate (toxic, serious health hazard, corrosive) and sodium borohydride (toxic, serious health hazard, corrosive, flammable) are used and are not suitable for high school students. After the synthesis, the product is washed several times and purified from

harmful residues. During the integration of the sensing material into the PDMS, methanol (flammable, toxic, serious health hazard) is used as a liquid to distribute the detection material well in the PDMS kit. However, this evaporates completely during the curing process of the film. After the whole process, the films can be used by students without any risk.

MEASUREMENTS AND CHARACTERIZATION

Hydrogen Measurements:

For hydrogen measurements, a 100 µL aliquot from the vial headspace was injected into a Shimadzu Nexis GC-2030 equipped with an A5 sieve column and BID detector. Detected hydrogen was compared against a calibration curve generated using 0,1% (All in gas), 1% (Nippon gases), and 3 % (Nippon gases) hydrogen synthetic air standards. The results were calculated in mmol/L (Tab. S1)

Sample 1 A/B/C: Illumination for 30 min with UV-flashlight (λ=395 nm); 3 mL EDTA solution (c= 4.9 mM), 5 mg platinum on alumina fiber (5% Pt), 4 mg PCNs

Sample 2 A/B/C: Illumination for 30 min with a blue flashlight; 3 mL stock solution (EDTA 4.9 mM, proflavine 0.05 mM), 5 mg platinum on alumina fiber (5% Pt), 4 mg PCNs

Sample 3 A/B/C: Illumination for 30 min with a blue flashlight; 3 mL EDTA solution (c= 4.9 mM), 5 mg platinum on alumina fiber (5% Pt), 4 mg PCNs

Table S1. Gas chromatographic measurements of samples 1-3 in different approaches

	$c(H_2)$ in mmol/L
**Sample 1A**	0.018
**Sample 1B**	0.085
**Sample 1C**	0.165
**Sample 2A**	0.057
**Sample 2B**	0.059
**Sample 2C**	0.064
**Sample 3A**	0
**Sample 3B**	0
**Sample 3C**	0

UV-Vis Spectroscopy

The samples were measured using a Varian Cary 4000 UV-Vis spectrophotometer, equipped with an integrating sphere (Ulbricht sphere). Reflectance measurements were performed using magnesium oxide (MgO) as the white reference standard. The measurements were conducted over a wavelength range from 800 nm to 200 nm, with data points recorded at 1 nm intervals. Notably, the spectrophotometer automatically switched its light source at 350 nm to optimize measurement accuracy. Measurements were taken with three different syntheses of polymeric carbon nitrides (Exp. 1, Fig. S2). These were then transferred to a Tauc plot to determine the size of the band gap (Fig. S3, Tab. S2). The maximum absorption ranged from 260 nm to 393 nm (Fig. S2), and a band gap of 2.78 eV was calculated from the Tauc plot (Fig. S3) which are consistent with values reported in the literature.[3,4]

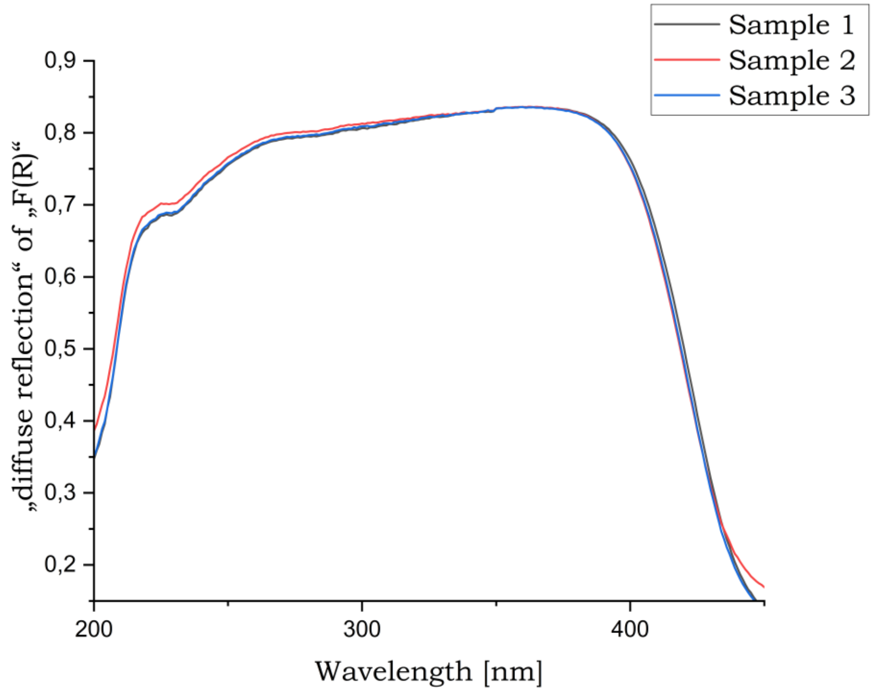

Figure S2. UV-Vis spectra of three different samples of polymeric carbon nitrides.

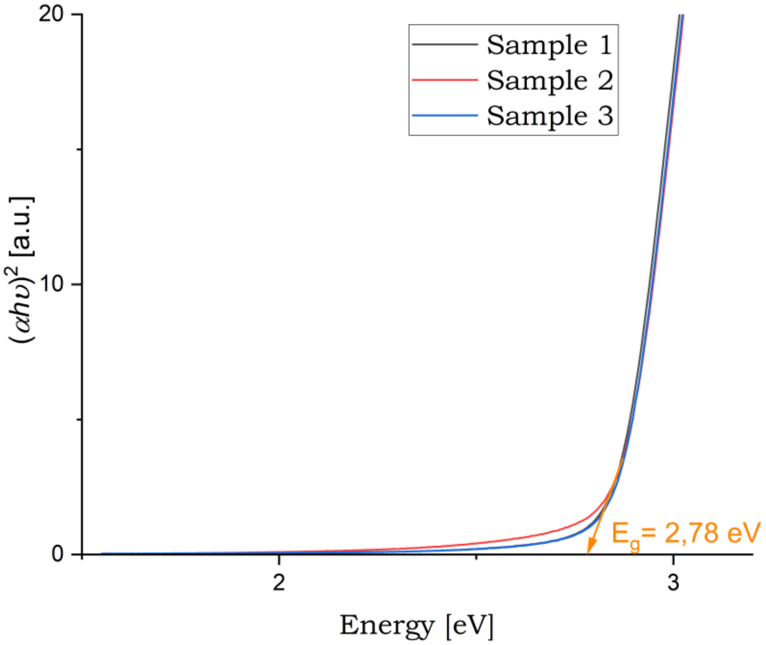

Figure S3. Determination of the band gap of three different samples via a Tauc plot.

Table S2. Calculated band gaps from the Tauc plot

	Band gap
**Sample 1**	2.78 eV
**Sample 2**	2.77 eV
**Sample 3**	2.72 eV

XRD-measurement

The X-ray powder diffraction patterns were obtained using a PANalytical Empyrean Series 2 diffractometer, utilizing Cu-Kα radiation (λ = 0.154 nm) from a copper anode. To reduce Kβ radiation, nickel filters were applied, and Soller slits with a width of 0.04 rad were used to minimize diffuse scattering. The data were recorded over a 2θ range of 5–80° in Bragg-Brentano geometry (θ-θ scan), with a step size of 0.013° and a measurement time of 159 s per step. A PIXcel 1D detector was used to capture the signal. Measurements were taken with three different syntheses of polymeric carbon nitrides. Peaks were observed at 27° and 13° (Exp. 1, Fig. S4, Tab. S3) and are consistent with values reported in the literature [3,4].

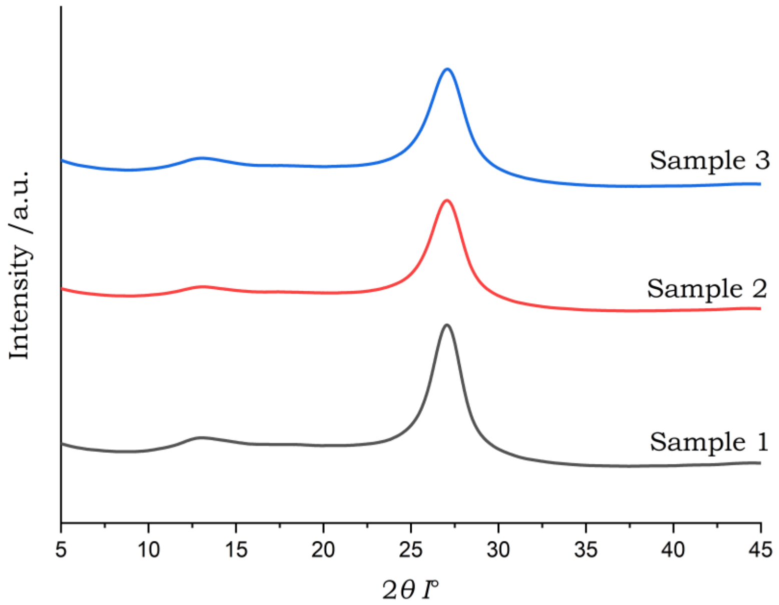

Figure S4. XRD measurement of three different samples of polymeric carbon nitrides.

Table S3. Peaks of the XRD-measurement.

	Peak 1	Peak 2
**Sample 1**	13.23	27.06
**Sample 2**	13.24	27.06
**Sample 3**	13.15	27.10

INTERVIEWS

Selection of candidates

We conducted a short interview with four students after the experiment. The aim of this was to get directly an impression about the feasibility of the experiment (Tab. S4) and what they felt was the most important topic they had learned (Tab. S5). Two students were selected from the upper quartile and two from the lower quartile, with the quartiles determined based on the mean value of items 1-3 (Publication, Fig. 8).

Results

Table S4. Feedback from the interviews on the feasibility of the experiment

	Answer (original in German)
**Candidate 1**	I actually got on relatively well with the implementation. I really have to say that, because I think everything that you have to do was described. The individual steps, what you have to add, how much. So in that respect, um, it was good. Everything was actually easy to understand. I mean, it's also written there. Shake, place on the UV flashlight, switch on, leave to stand etc. There was nothing that was somehow incomprehensible or that should or could have been described more.
**Candidate 2**	Yes, everything actually worked quite well. I can't say much about it now. Um. It was actually relatively well structured and it is also well presented in the booklet what you should do and also yes (...) I actually had few problems.
**Candidate 3**	So the implementation/ because everything was already in place and it was really easy. You gave great instructions. We knew straight away how to turn it up. It's about the three little ones now, isn't it? [...] Okay. Yes. No. But you also knew from the pictures roughly how much of the spatula/ So how much/ What size of spatula tip to use. So it was really, I would say foolproof.
**Candidate 4**	Yes, I generally got on very well with it. Everything was well explained in these booklets. What was a bit difficult, for example, was getting the hydrogen into the glass from the tube. That was a bit time-consuming. But otherwise I think/ Yes. It actually went quite well.

Table S5. Feedback from the interviews about the most important topic they have learned from the experiment

	Answer (original in German)
**Candidate 1**	Mhm. I actually think that/what different areas of application there are. Because I mean, you don't really hear anything about the subject at school. Except maybe once in photosynthesis. There was a brief mention somewhere about light and things like that, but um, that you can actually do so much with it and what different possibilities there are, not just water purification, for example, but also hydrogen production. All kinds of things. These are things that you don't even think about. Because I mean, when we look at hydrogen production at school, we kind of look at things like electrolysis and fuel cells. So, um, but somehow you don't know that at all and I think that's really interesting and important, because you only ever hear that physics is important for the future, but with chemistry it's always like this, well/
**Candidate 2**	Yes (...) simply what can happen, what light, UV light can do in the end/ So all in all, what was interesting about the whole two days, what you can do/ You don't realize that in normal life, what you can do with UV light and so on. That was actually the most interesting thing.
**Candidate 3**	Well, on the one hand, that there is this foil that can prove it and on the other hand, I didn't know. I didn't know that platinum played such a big role. In the whole thing. I found that really amazing.
**Candidate 4**	Just with the ethanol (*comment: in an earlier version, the students worked with ethanol instead of EDTA as an electron donor*). That hydrogen is produced from it. Personally, I would have liked to have seen a bit more information on how the ethanol is turned into hydrogen. Where does it come from? That would have been quite good. But yes, that was interesting.

AUTHOR INFORMATION

Corresponding Author

*E-mail: malte.petersen2@uol.de, timm.wilke@uol.de

ACKNOWLEDGMENTS

The authors Petersen, Bauschulte, Hotzel, Peneva and Wilke are grateful for funding this work through the Collaborative Research Center/Transregio 234 CataLight, project number 364549901 (projects Ö1 and A3) from the German Research Foundation (DFG).

The authors Talledo and Bernhard are grateful for the financial support from the US National Science Foundation (CHE-2350257).

The author Wark thanks the Federal Ministry of Education and Research for financial support within the project PRODIGY (grant number 033RC024B).

REFERENCES

(1) Vorontsov, A. V.; Stoyanova, I. V.; Kozlov, D. V.; Simagina, V. I.; Savinov, E. N. Kinetics of the Photocatalytic Oxidation of Gaseous Acetone over Platinized Titanium Dioxide. *Journal of Catalysis* **2000**, *189* (2), 360–369. DOI: 10.1006/jcat.1999.2717.

(2) He, Z.; Xie, L.; Tu, J.; Song, S.; Liu, W.; Liu, Z.; Fan, J.; Liu, Q.; Chen, J. Visible Light-Induced Degradation of Phenol over Iodine-Doped Titanium Dioxide Modified with Platinum: Role of Platinum and the Reaction Mechanism. *J. Phys. Chem. C* **2010**, *114* (1), 526–532. DOI: 10.1021/jp908946c.

(3) Wen, J.; Xie, J.; Chen, X.; Li, X. A review on g-C 3 N 4 -based photocatalysts. *Applied Surface Science* **2017**, *391*, 72–123. DOI: 10.1016/j.apsusc.2016.07.030.

(4) Weers, M.; Seggern, A. R. von; Vocke, H.; Taffa, D. H.; Wark, M. Two Ways to more NH 2 -Groups: Formation of Polymeric Carbon Nitride via Melem Tetramer Nano Sheets or Supramolecular Assembly of Melamine and Cyanuric Acid for Applications as Photocatalyst. *ACS Appl. Nano Mater.* **2024**, *7* (1), 1402–1415. DOI: 10.1021/acsanm.3c05516.
