# Supplementary material for: Synthesis of Polymeric Carbon Nitrides in a Low-Cost Moka Furnace for Photocatalytic Hydrogen Generation with Visible Light
Source: J Chem Educ. 2025 Jun 3;102(7):2912–9. doi: 10.1021/acs.jchemed.5c00114 (PMC12243077; doi:10.1021/acs.jchemed.5c00114)
Supplement: Supplementary file 3 [file ed5c00114_si_004.docx]

**Supporting Information – Student Handouts**

**Synthesis of Polymeric Carbon Nitrides in a low-cost Moka Furnace for Photocatalytic Hydrogen Generation with Visible Light**

Malte Petersen^1,*^, Jonathan Bauschulte^1^, Savannah Talledo^2^, Konrad Hotzel^3,4^, Michael Wark^5^, Kalina Peneva^4,6,7^, Stefan Bernhard^2^, Timm Wilke^1,6,*^

^1^ Institute of Chemistry, Chemistry Education, Carl von Ossietzky University Oldenburg, Carl-von-Ossietzky-Str. 9-11, 26129 Oldenburg, Germany

^2^ Department of Chemistry, Carnegie Mellon University, 4400 Fifth Avenue, Pittsburgh, Pennsylvania 15213, USA

^3^ Center for Energy and Environmental Chemistry Jena II (CEEC Jena II), Friedrich-Schiller-University Jena, Lessingstraße 12, 07743 Jena, Germany

^4^ Institute of Organic Chemistry and Macromolecular Chemistry, Friedrich Schiller University Jena, Lessingstraße 8, 07743 Jena, Germany

^5^ Institute of Chemistry, Chemical Technology 1, Carl von Ossietzky University Oldenburg, Carl-von-Ossietzky-Str. 9-11, 26129 Oldenburg, Germany

^6^ Jena Center of Soft Matter, Friedrich-Schiller University Jena, Philosophenweg 7, 07743 Jena, Germany

^7^ Center for Energy and Environmental Chemistry Jena (CEEC Jena), Friedrich-Schiller University Jena, Philosophenweg 7a, 07743 Jena, Germany

AUTHOR INFORMATION

Corresponding Author

*E-mail: malte.petersen2@uol.de, timm.wilke@uol.de

Acknowledgments

The authors Petersen, Bauschulte, Hotzel, Peneva and Wilke are grateful for funding this work through the Collaborative Research Center/Transregio 234 CataLight, project number 364549901 (projects Ö1 and A3) from the German Research Foundation (DFG).

The authors Talledo and Bernhard are grateful for the financial support from the US National Science Foundation (CHE-2350257).

The author Wark thanks the Federal Ministry of Education and Research for financial support within the project PRODIGY (grant number 033RC024B).

Photocatalysis

student laboratory

The potential of nanomaterials in the future

| Date: |  |
| --- | --- |
| Name: |  |

Welcome to the student laboratory about **photocatalysis**

In this student laboratory, we want to investigate the potential of nanomaterials for the future. We will focus on the topic of light-driven reactions as a sub-aspect of nano chemistry. In two theory stations, we will lay the foundation for the experiments and then investigate how nanomaterials can be used in photocatalytic hydrogen production.

# Table of contents

| Page | Title |
| --- | --- |
| 4 | T1 - Synthesis of Polymeric Carbon Nitrides (PCNs) |
| 7 | T2 - What is a (Photo)-catalyst? |
| 9 | E1 - The Hydrogen Detection Film |
| 11 | E2 - Photocatalytic Hydrogen Generation |
| 13 | E3 - Photosensitized Hydrogen Generation |

# T1 - Synthesis of Polymeric Carbon Nitrides (PCNs)

**Polymeric carbon nitrides** (PCNs) are a class of materials consisting of carbon and nitrogen atoms. These materials are structured like **polymers**, which means that they consist of long chains of repeating units connected by covalent bonds. Due to their small size, PCNs are classified as **nanomaterials**. A distinction is made between the “bottom-up” and “top-down” methods for synthesizing PCNs and other nanomaterials. Their principles are illustrated in the following figure.


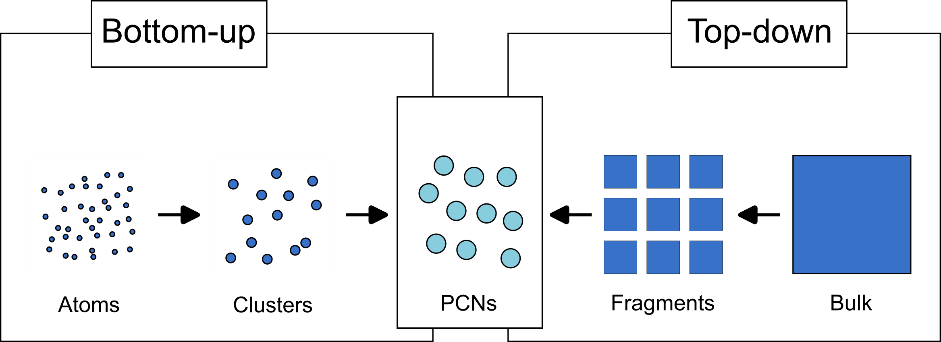


**A1**: Describe the difference between “top-down” and “bottom-up” procedures using the figure above.

|  |
| --- |
|  |
|  |
|  |
|  |

**A2**: Assign the following methods for synthesizing nanostructures to the “bottom-up” or “top-down” mechanism.

|  |  |  |  |  | |
| --- | --- | --- | --- | --- | --- |
| **Electrochemical**  **oxidation** |  | **Microwave synthesis** |  | **Hydrothermal**  **treatment** | |
| Electrolysis causes parts of the carbon electrodes to decompose and enter the electrolyte solution. |  | A microwave reactor causes carbon-based particles to react. Larger units such as PCNs are formed. |  | A solution is heated over a longer period and the carbon-based particles assemble into larger units. | |
|  |  |  |  |  | |
|  |  |  |  |  | |
| **Thermal treatment** |  | **Laser ablation** |  | **Chemical oxidation** | |
| Heating breaks the bonds between the particles and reassembles them into larger units. |  | Carbon materials are bombarded with a powerful laser, causing smaller units to split off. |  | The carbon-based materials are broken down into smaller components by a strong oxidizing agent. | |
| Introduction | | | | |  |


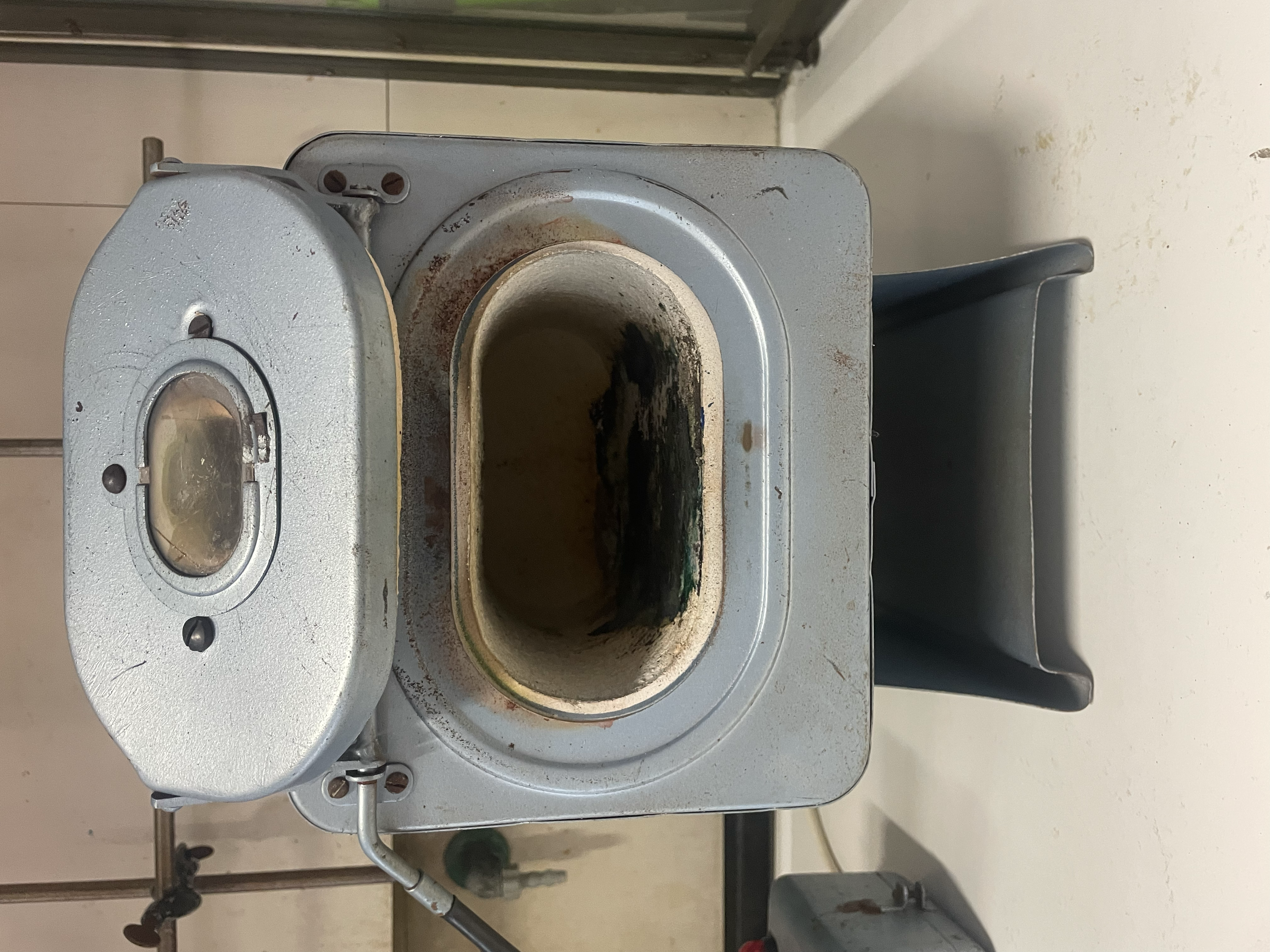
After presenting a wide range of synthesis possibilities for nanostructures, we will now focus on polymeric carbon nitrides. A simple and frequently used synthesis is the **heating** of urea in a **muffle furnace** (picture on the right). A muffle furnace is constructed in a similar way to a household furnace. However, it can reach significantly higher temperatures of **up to 1,300 °C** and keep them constant.

| Materials | Chemicals | GHS |
| --- | --- | --- |
| - Ultraviolet flashlights | - Polymeric carbon nitrides - Urea [1] | 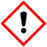[1] |

| Task |
| --- |

**Compare the optical properties of the urea and the polymeric carbon nitrides under ambient light and UV light!**

| Observations | | |
| --- | --- | --- |
|  | **Urea** | **Polymeric carbon nitrides** |
| **Ambient light** |  |  |
| **UV light** |  |  |

| Evaluation |
| --- |

**Polycondensations** and **polymerizations** take place when **urea** is heated. Different temperatures lead to the formation of various intermediate products. At a temperature below 240 °C, **melamin** is formed from urea. This reacts further at temperatures of 390 °C to form **melem**. This is followed by condensation to form **melon**. From a temperature of 520 °C, our product forms **polymeric carbon nitrides**. The PCNs are characterized by their yellowish-white color and solid-state luminescence.

**A3:** With the help of the evaluation text, label the following molecules and arrange the names in the correct order. Also write down the temperatures required for the individual steps.

**Temperature**:
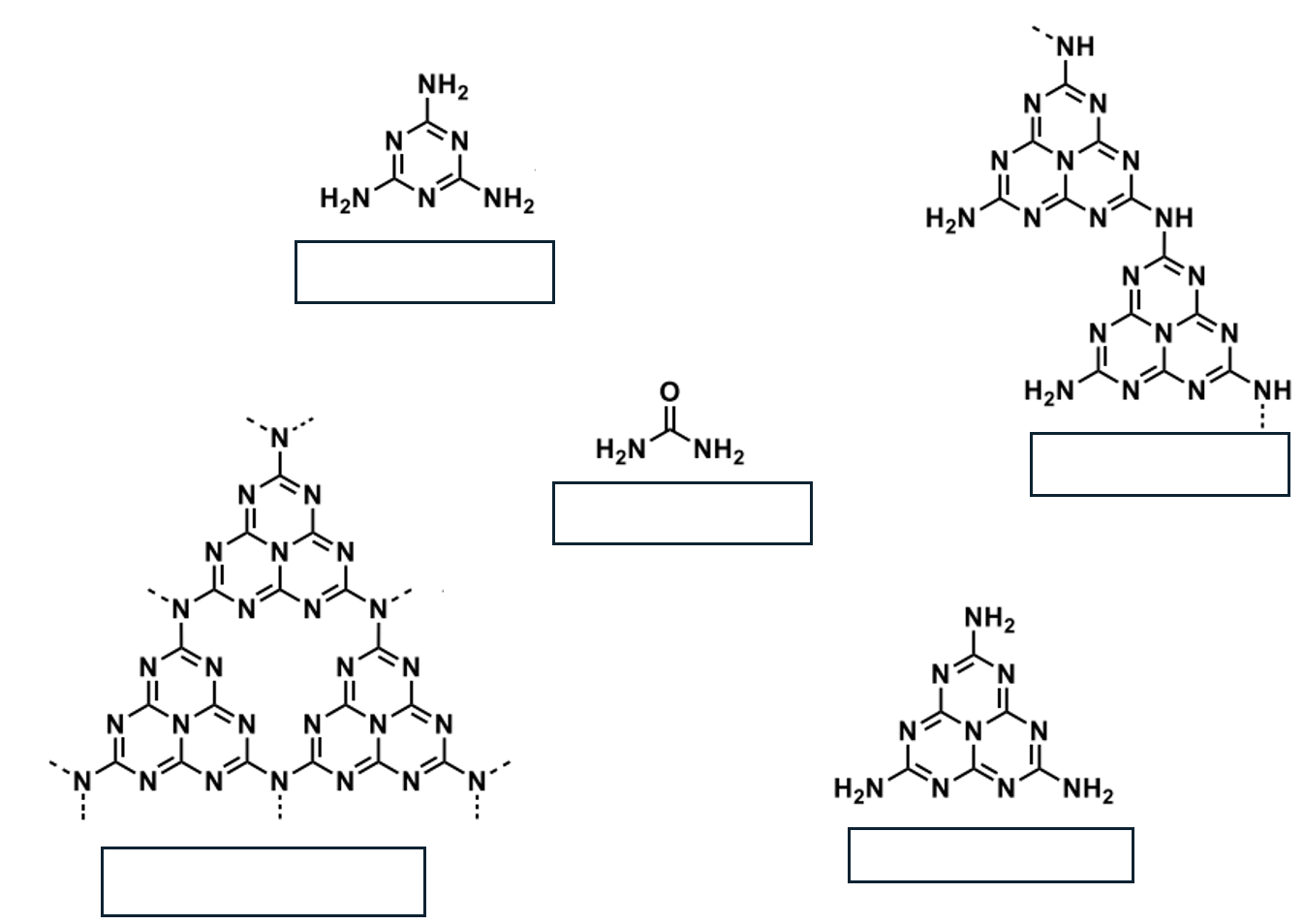
**390 °C >520 °C <240 °C**

**Reaction:**

**A4:** Explain which synthesis method this experiment can be assigned to.

|  |
| --- |
|  |
|  |
|  |
|  |
|  |

# T2 - What is a (Photo)-catalyst?

**Catalysts** are substances that are added to reactions in order to initiate them or increase their efficiency. They reduce the **activation energy** of the reaction and enable a reaction to start with less energy input. Nevertheless, they have **no influence on the equilibrium** of the chemical reaction and emerge from the reaction **unchanged**.


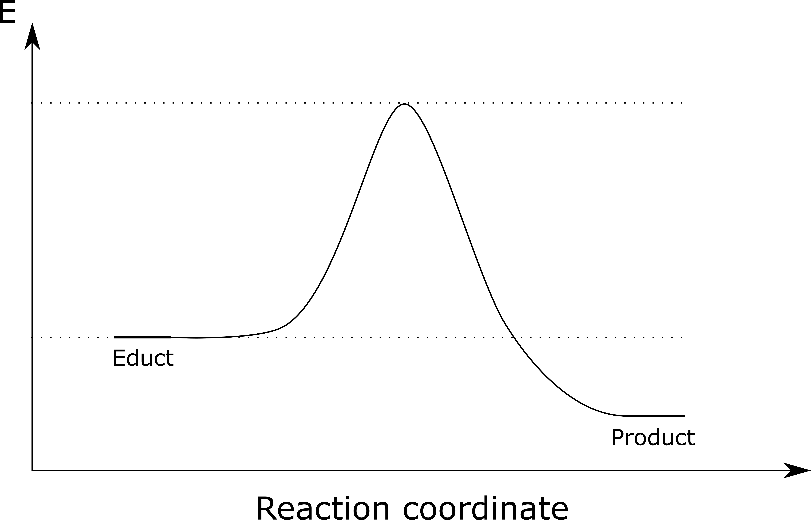
**A1:** Draw the course of a catalyzed reaction in the figure below. For comparison, the course of a non-catalyzed reaction is already shown.

A distinction is made between **homogeneous** and **heterogeneous** catalysts. Homogeneous catalysts are in the same aggregate state as the reactants. For example, sulphuric acid is used as a catalyst in esterification, i.e. the reaction of a carboxylic acid and an alcohol. Heterogeneous catalysts are present in a different aggregate state, as in the exhaust catalyst in a car.

**But what is a photocatalyst?**

The term **photocatalyst** is a combination of the word photon (from the ancient Greek φῶς phōs “light”) and catalyst. As the word suggests, photocatalysts are substances that can catalyze a chemical reaction by absorbing light. Just as with the already known classic catalysts, the photocatalyst emerges unchanged from this process and can be divided into the subgroups homogeneous and heterogeneous.

| 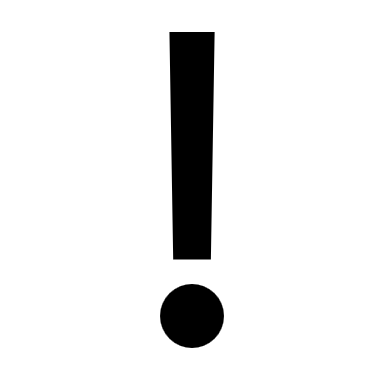 | A photocatalyst is a substance that enables a chemical reaction when exposed to light. It emerges unchanged from this reaction. |
| --- | --- |

**Mechanism of heterogeneous photocatalysis**

**A2**: Label the following boxes with the help of the information text.


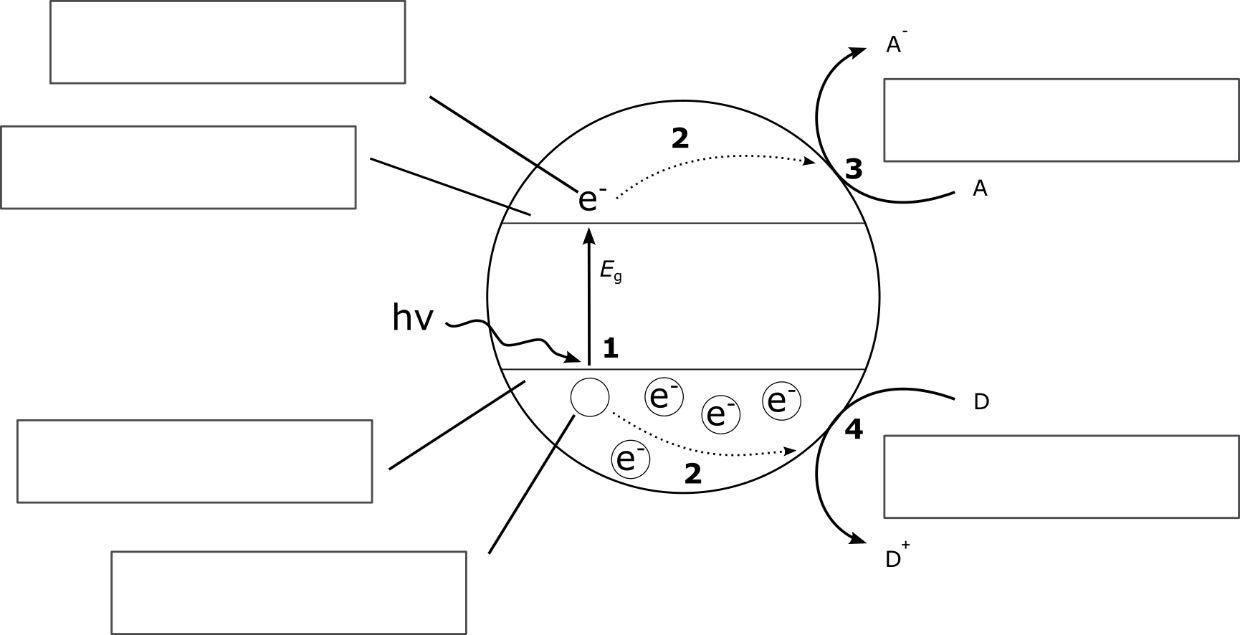


The mechanism of heterogeneous photocatalysis is based on the following mechanism. When light (*hv*) from the **ultraviolet** spectrum hits titanium dioxide, the **photons** in it can excite an **electron** with their momentum and lift it out of the **valence band** (VB) into the **conduction band** (CB) (1). This results in the formation of so-called electron-hole pairs (**e^-^/h⁺**). By **lifting** the electron into the conduction band, an **electron deficit** remains in the crystal structure of the substance, which we can imagine in the following simplified form as a hole (**h^+^**) where an electron (**e^-^**) has been knocked out. Both the hole in the valence band and the electron in the conduction band are **highly reactive** and can be moved to the outer surface of the substance by **charge transport** (2). There they can react with other substances and initiate a **reduction** (3) (in the case of the electron) or an **oxidation** (4) (in the case of the electron hole).

# E1 - The Hydrogen Detection Film

| Introduction |
| --- |

The oxyhydrogen sample is typically used to detect hydrogen. However, this is not suitable for such small quantities as those formed in photochemical experiments. In addition to the oxyhydrogen sample, there is also another way of detecting hydrogen.

| Task |
| --- |

**Investigate what effect different gases have on the detection foil!**

| Materials | Chemicals | GHS | |
| --- | --- | --- | --- |
| - 3 glass vials with lids | - Gas cylinders - Oxygen ^[2, 3]^ - Carbon dioxide ^[1, 2]^ - Hydrogen ^[2]^ - 3 pieces of detection foil | 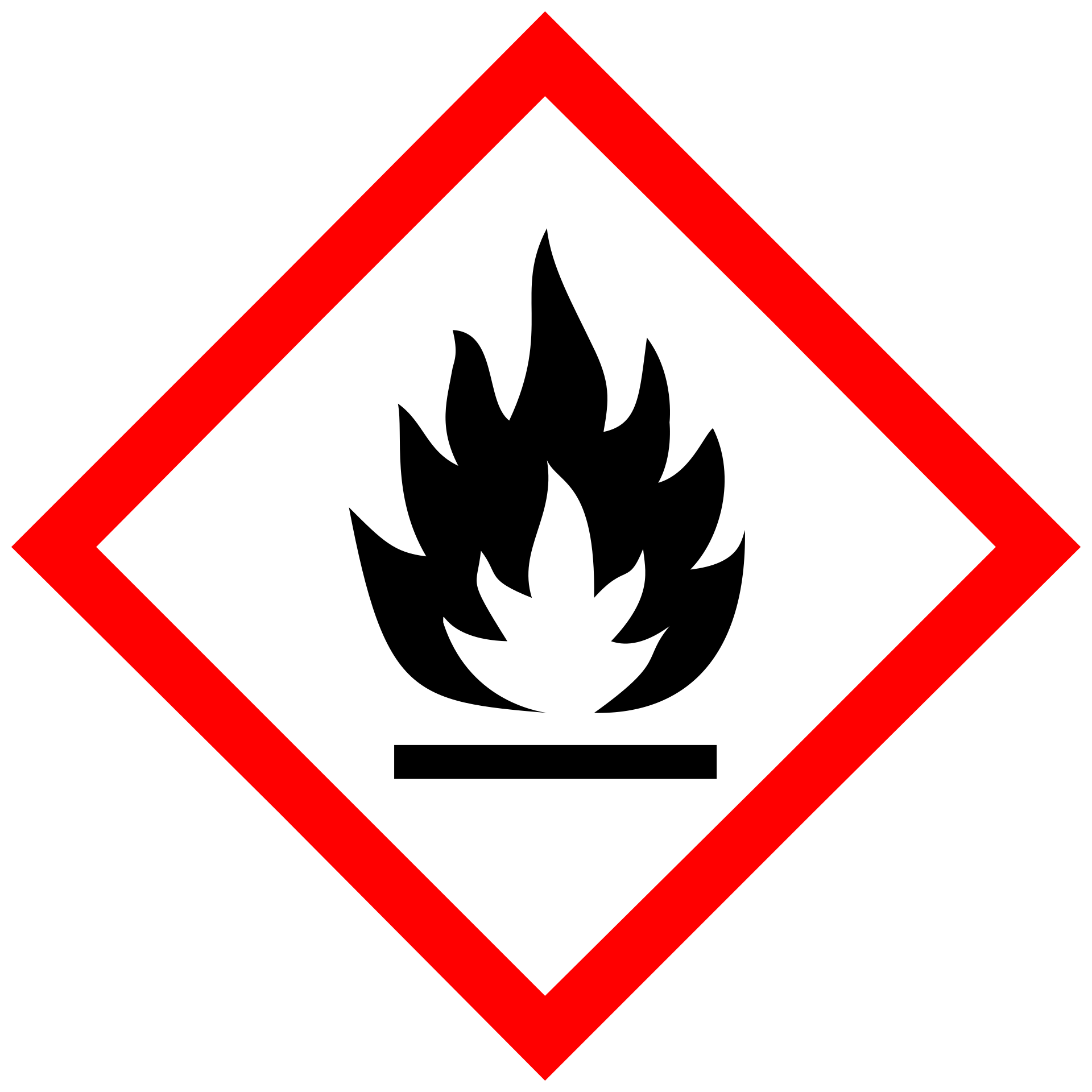[1] | 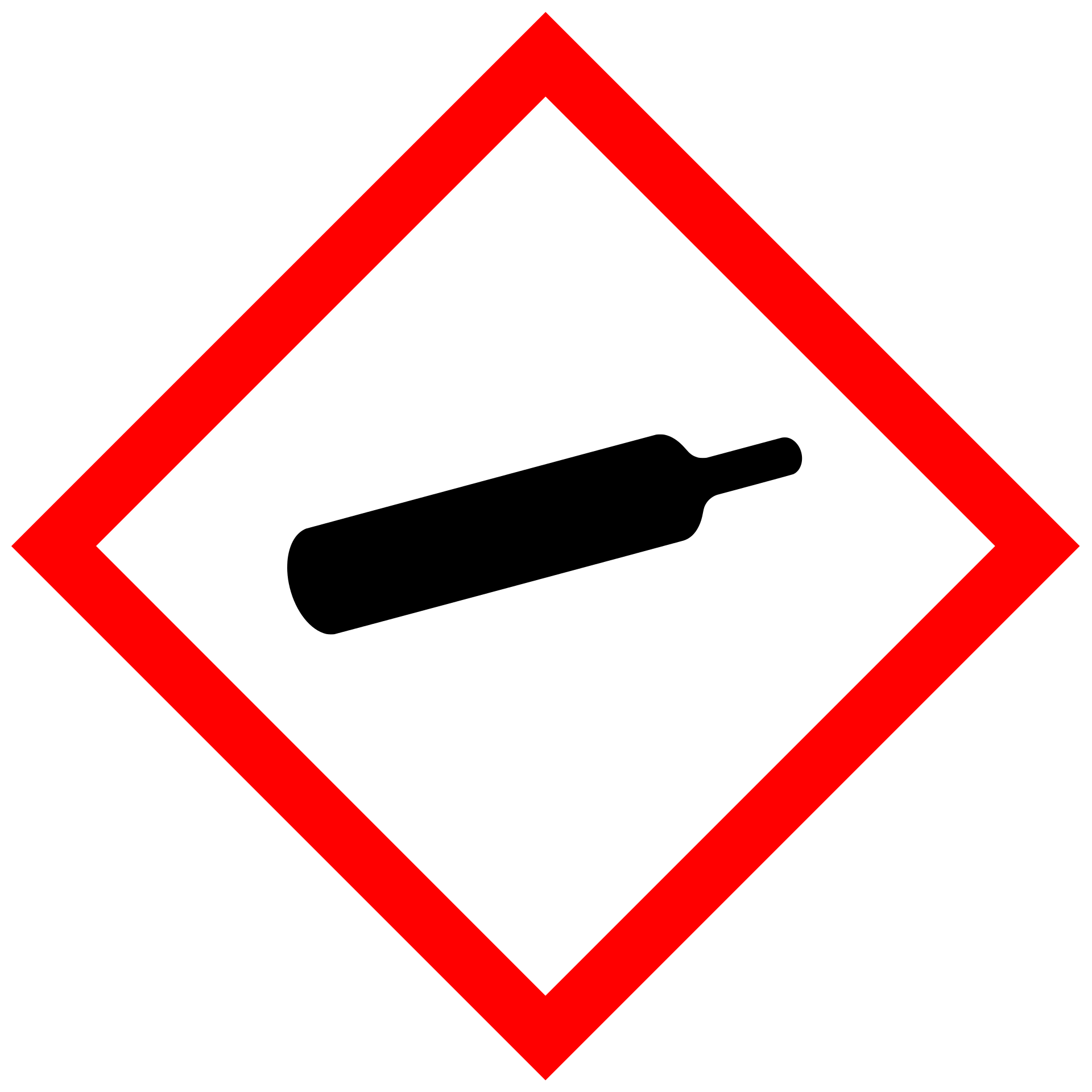[2] |
|  |  | 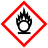[3] |  |

| Procedure |
| --- |

1. Put a piece of foil in each of the three glass vials and label them with the respective gases.
2. Introduce oxygen, carbon dioxide and hydrogen into the corresponding vial. Close the vials immediately after introduction.
3. Observe the foils for a few minutes.

| Observation |
| --- |

|  |
| --- |
|  |
|  |
|  |
|  |
|  |
|  |

## Evaluation

| Information: Why does the foil change color? |
| --- |
| 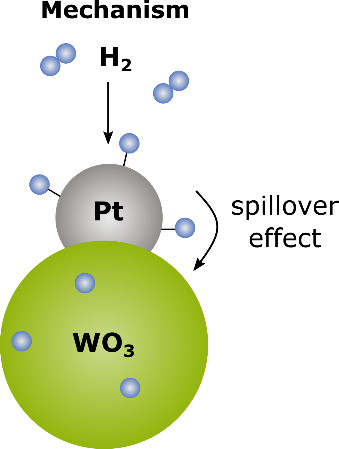The detection foil contains **platinum (Pt)** and **tungsten trioxide (WO_3_)**. Platinum serves as a catalyst, as the hydrogen molecules can be adsorbed on its surface. These are split into **protons** and free **electrons** and transferred to the tungsten trioxide molecules through the so-called “spillover effect”. The result is **tungsten bronze**, which has a **dark blue color**. In our case, the molecule is represented in simplified symbolic notation as **HWO_3_**. |

**A1:** Write the two reaction equations for the formation of tungsten bronze.

Equation 1:

|  |
| --- |

Equation 2:

|  |
| --- |

*Hint: Equation 1: Platinum is used catalytically to form protons and electrons.*

**A2:** After a few hours in the air, the film turns yellow again and becomes slightly moist. Explain which substances are formed during the reaction and establish the corresponding reaction equation.

| 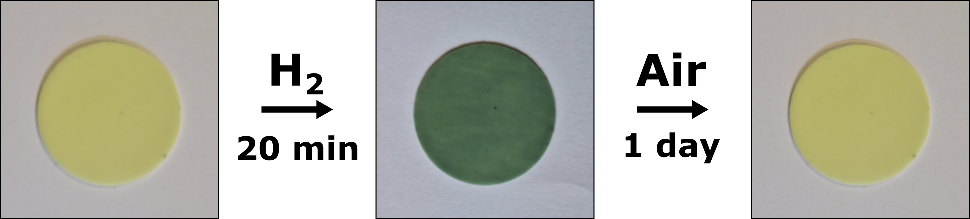 |
| --- |
|  |
|  |
|  |
|  |

Equation 3:

*Hint: Which molecule from the air could the tungsten bronze react with?*

|  |
| --- |

# E2 - Photocatalytic Hydrogen Generation

| Introduction |
| --- |

**Hydrogen** plays an important role as a renewable **energy source** and could play a key role in energy supply and storage in the future. It can be produced by the **electrolysis** of water, but this process requires a lot of energy and electricity. An alternative method is **photocatalytic hydrogen production**, which uses sunlight as the necessary energy source. Typically, semiconductors consisting of transition metals are used for this, but other types of material also look promising.

| Task |
| --- |

**Check whether the polymeric carbon nitrides can form hydrogen!**

| Materials | Chemicals | GHS |
| --- | --- | --- |
| - Glass vial with lid - Spatula - UV-flashlight - Detection foil | - Polymeric carbon nitrides (PCNs) - EDTA-solution (c = 4,9 mM) ^[1]^ - Platinum on alumina fiber | 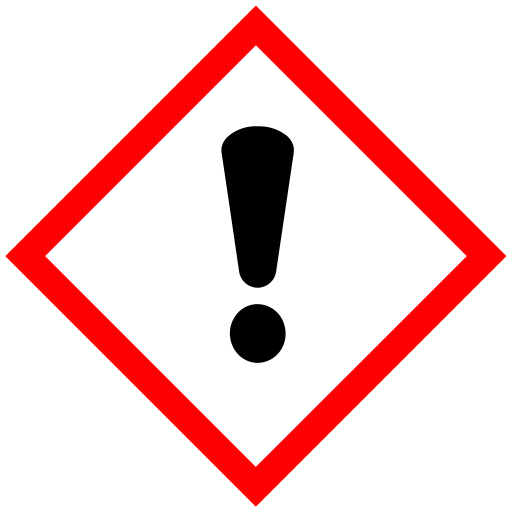[1] |

| Procedure |
| --- |

1.
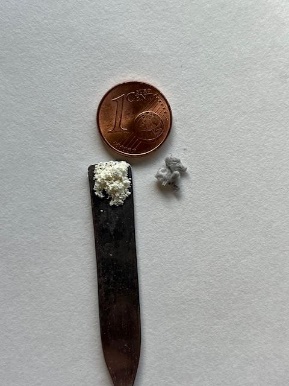
Add 3 mL EDTA solution, a spatula tip of the PCNs, a spatula tip of aluminized platinum and a piece of the detection foil (see right) to a glass vial.
2. Close the vials and shake it until the foil settles on the bottom.
3. Place the snap lid jar on the UV flashlight and illuminate it for 15 to 30 minutes. Then examine the foil for its coloration.

| Observation |
| --- |
|  |
|  |
|  |
|  |
|  |

## Evaluation

**A1**: Using your observations and your knowledge of the film, explain how to interpret the blue coloration.

|  |
| --- |
|  |
|  |

| Information: Photocatalytic hydrogen generation |
| --- |
| As already discussed in the theory station on photocatalysis, it is possible to initiate oxidative or reductive reactions by generating **electron-hole pairs**. In hydrogen production, protons are **reduced** to hydrogen by electrons in the **conduction band**. In contrast, the so-called electron donor EDTA is **oxidized** to EDTA^+^ in the **valence band**. As the polymeric carbon nitrides do not provide suitable active centers for the formation of hydrogen, platinum is added as a co-catalyst. |

**A2:** Set up the partial equations and then the redox reaction.

Reduction:

|  |
| --- |

Oxidation:

|  |
| --- |

Redox reaction:

|  |
| --- |

*Hint: Two electrons are required for reduction and one hole for oxidation.*


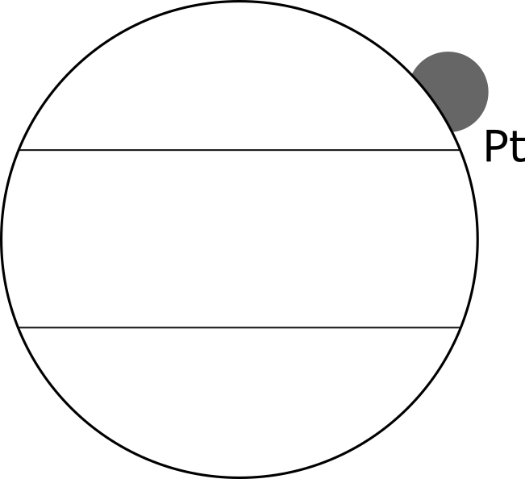
**A3**: Draw the mechanism for photocatalytic hydrogen production in the following figure and label it.

*Hint: If in doubt, take another look at the theory station on photocatalysis.*

# E3 - Photosensitized Hydrogen Generation

| Introduction |
| --- |

We have already learned that UV light can be used to drive photochemical reactions. However, current research is increasingly investigating how a broader spectrum of light can be used. So-called **photosensitizers** are used for this purpose.

| Task |
| --- |

**Investigate how the photosensitizer works!**

| Material | Chemicals | GHS |
| --- | --- | --- |
| - Glass vial with lid - Spatula - Blue-flashlight - Detection foil - Tripod with clamps | - Polymeric carbon nitride (PCNs) - Stock-solution (EDTA (c=5 mM) ^[1]^ ,   Proflavin (c=0,05 mM) ^[1]^)   - EDTA-solution (c=5 mM) ^[1]^ - Platinum on alumina fiber | 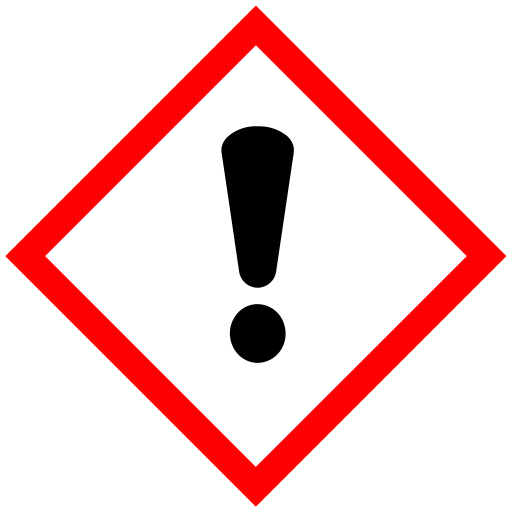[1] |

| Procedure |
| --- |

1.
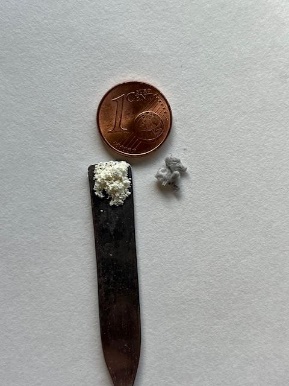
Add 3 mL EDTA solution, a spatula tip of the PCNs, a spatula tip of aluminized platinum and a piece of the detection foil (see right) to a snap lid vial.
2. Add the stock solution to the second snap lid vial instead of the EDTA solution.
3. Close the snap lid vials and shake them until the foil settles to the bottom.
4. Clamp the snap-on lid vials over the blue flashlight.
5. Switch on the light to stop the time. The lighting time is 15 to 30 minutes.

| Observation |
| --- |
|  |
|  |
|  |
|  |
|  |
|  |
|  |

## Evaluation

To understand why we were able to visualize hydrogen by illuminating it with blue light, we must first look at the mechanism of fluorescence. This will help us to understand the actual mechanism.

**A1**: Explain the mechanism of fluorescence and draw a supporting illustration.

|  |
| --- |
|  |
|  |
|  |
|  |
|  |

Drawing area

| Information: Photosensitizer |
| --- |
| A photosensitizer (PS) is a substance that enables chemical reactions when exposed to light. These molecules absorb light energy, whereby an **electron** is **raised** from the highest occupied orbital (Highest Occupied Molecule Orbital - HOMO) to the lowest unoccupied orbital (Lowest Unoccupied Molecular Orbital - LUMO). In contrast to fluorescence, the electron does not fall back to the ground state but can be transferred to another molecule. |

**A2**: Explain how a possible mechanism of a photosensitized reaction works. Use the diagram shown and add the important elements. (Advanced)


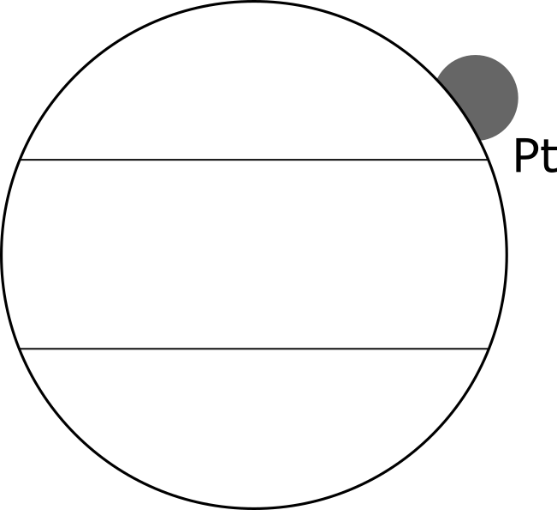


|  |
| --- |
|  |
|  |
|  |
|  |
|  |
|  |

*Hint: The electron is transferred from the LUMO of the light collector to the conduction band of the PCNs. It is important here that the LUMO must be higher in energy than the conduction band.*

**A3:** Explain why the foil did not change color in the sample without the light collector.

|  |
| --- |
|  |
|  |
|  |
|  |
|  |
